# Niche‐Mediated Neural Priming Enables Robust and Scalable Generation of Human Choroid Plexus Organoids

**DOI:** 10.1002/advs.77512

**Published:** 2026-08-31

**Authors:** Zhongliang Chen, Liangxia Jiang, Xianbin Wang, Lili Zhong, Rongcan Luo, Min Su, Liangzhao Chu, Peng Luo

**Affiliations:** ^1^ Center For Tissue Engineering and Stem Cell Research Translation Medicine Research Center Guizhou Biomanufacturing Laboratory Guizhou Medical University Guiyang China; ^2^ Department of Neurosurgery The Affiliated Hospital of Guizhou Medical University Guiyang China; ^3^ Guizhou Provincial Key Laboratory of Pathogenesis and Prevention of Common Chronic Diseases Guizhou Medical University Guiyang China; ^4^ Department of Physiology & Pathophysiology School of Basic Medicine Guizhou Medical University Guiyang China; ^5^ Department of Rehabilitation Medicine The Affiliated Hospital of Guizhou Medical University Guiyang China; ^6^ Department of Rehabilitation Medicine Clinical Medical College Guizhou Medical University Guiyang China; ^7^ Department of Endocrinology Central Hospital of Wuhan Tongji Medical College Huazhong University of Science and Technology Wuhan China; ^8^ Gansu Key Laboratory of Biomonitoring and Bioremediation for Environmental Pollution, and Ministry of Education Key Laboratory of Cell Activities and Stress Adaptations School of Life Sciences Lanzhou University Lanzhou China; ^9^ Key Laboratory of Environmental Pollution Monitoring and Disease Control Ministry of Education School of Public Health Guizhou Medical University Guiyang China; ^10^ Collaborative Innovation Center For Prevention and Control of Endemic and Ethnic Regional Diseases Co‐Constructed By the Province and Ministry Guizhou Medical University Guiyang China

**Keywords:** choroid plexus, embryonic stem cells, neural priming, neuroepithelial cells, niche, organoids

## Abstract

The choroid plexus (ChP) secretes cerebrospinal fluid (CSF) and forms the blood‐CSF barrier (BCSFB), playing indispensable roles in brain development and homeostasis. However, tractable human ChP models remain limited, with current organoid protocols largely overlooking the stem cell niche as a determinant of lineage commitment. Here, we report that a small number of ChP organoids sporadically emerge during skin organoid induction, and switching human embryonic stem cells (hESCs) culture from E8 to mTeSR1 medium—preconditioning cells toward a neural‐primed state—substantially enhances ChP organoid induction efficiency from <3% to ∼68%. Mechanistically, mTeSR1 culture suppresses BMP and WNT activity while upregulating neuroepithelial programs, thereby enabling robust ChP differentiation upon BMP4 exposure. The resulting organoids recapitulate key structural and functional attributes of native ChP, including polarized epithelium and CSF‐like fluid (iCSF) secretion. Notably, iCSF‐derived exosomes closely mirror the protein composition of native CSF exosomes. Furthermore, expression of the early ChP markers OTX2 and MSX1 suggest progressive specification toward the ChP fate. These ChP organoids likely represent a mixed ventricular identity with predominant hindbrain characteristics and exhibit selective susceptibility to SARS‐CoV‐2 infection, validating their physiological relevance. This niche‐driven priming strategy provides a scalable platform for modeling ChP development, barrier transport, and neurological disorders.

## Graphical abstract

Compared to E8, mTeSR1 culture downregulated WNT and TGF‑β signaling, and upregulated neural differentiation pathways, enhancing neural priming. Under skin organoid induction conditions, mTeSR1‑cultured hESCs generated organoids with significantly higher neuroepithelial differentiation. With the progressive expression of early ChP developmental genes, including OTX2 and MSX1, the organoids were gradually directed toward a ChP fate.

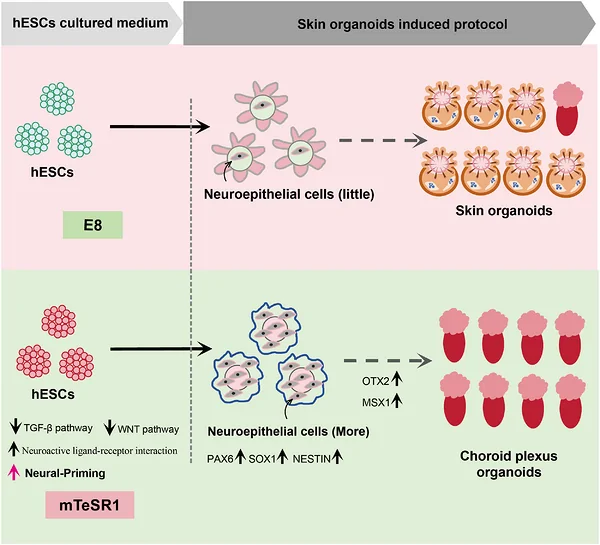

## 1 Introduction

The choroid plexus (ChP) comprises a specialized secretory epithelium that protrudes into each cerebral ventricle to form the morphological and functional core of the blood‐cerebrospinal fluid barrier (BCSFB) [1]. Enveloping a fenestrated capillary network and a collagen‐rich stroma, the epithelium expresses a variety of tight junction proteins, solute transporters, and generates >80% of the cerebrospinal fluid (CSF) [2]. Through directed ion transport, targeted release of growth factors, and controlled leukocyte trafficking, the ChP regulates neurogenesis, synaptic maturation, metabolic buffering, and immunosurveillance across the brain [3, 4]. Age‐related or disease‐driven perturbations of epithelial polarity, transporter expression, CSF secretion, or barrier integrity compromise ChP functions, resulting in various neurological disorders, such as Alzheimer's disease (AD) [5, 6]. Despite its physiological importance, the human ChP remains poorly characterized due to limited accessibility and scarcity of experimental models.

Following gastrulation, the embryonic surface forms a single layer of neuroectoderm, which will ultimately differentiate into the nervous system and skin epithelium [7]. Its developmental fate, however, is largely determined by WNT signaling, which inhibits the response of ectoderm to fibroblast growth factors (FGFs), and then promotes bone morphogenetic proteins (BMPs) expression, thereby directing epidermal fate [8, 9, 10]. Conversely, WNT inhibition activates FGF signaling, reduces BMP expression, and drives ectodermal cells toward neural crest lineages [11, 12]. Thus, precise regulation of FGF, BMP and WNT pathways enables the directed differentiation of neuroectoderm, neural crest, cranial placodes, and non‐neural ectoderm (e.g., skin) [13].

During nervous system development, the ectoderm forms the neural plate, which subsequently folds into the neural tube [2]. The neural tube closes at the dorsal midline, and the top of this tubular structure, known as the roof plate, serves as an important patterning center in the central nervous system (CNS). The roof plate secretes various signaling molecules, including BMPs and WNTs, and the balance of these signals determines tissue fate. Among them, BMP4 is indispensable for ChP development [2, 14], which can directly induce ChP epithelial differentiation [15, 16]. Adjacent to the ChP, the cortical hem increases the secretion of WNT signals as the ChP develops [17]. Based on these findings, pluripotent stem cells (PSCs) can be directed toward ChP epithelium or organoids in vitro by modulating BMP and WNT signaling. In 2020, two research groups independently reported the generation of ChP organoids. During forebrain organoids induction, Pellegrini et al. combined BMP4 and CHIR99021 (a WNT activator) treatment to obtain telencephalic ChP organoids with well‐defined boundaries and fluid‐filled compartments, recapitulating key morphological and functional features of human ChP [18]. By using a similar method, Jacob et al. also obtained ChP organoids, although these organoids did not form the fluid‐filled cavities [19].

In the present study, we observed sporadic formation of ChP organoids during skin organoid induction, albeit at a low proportion (<3%). The ChP epithelium derived from neuroepithelial cells is an important brain component. Given that both the brain and skin originate from the ectoderm, we hypothesized that, in the context of skin organoid induction, enhancing the neural differentiation efficiency of hESCs―particularly the proportion of neuroepithelial cells―might increase the formation rate of ChP organoids. Previous studies have indicated that human pluripotent stem cells (hPSCs) cultured in mTeSR1 exhibit a stronger priming tendency toward neural lineage differentiation [20, 21]. Surprisingly, when switching the hESCs culture medium from E8 to mTeSR1, we observed reduced BMP and WNT signaling activity together with a significant increase in neural priming, which promoted neuroepithelial differentiation and ultimately lead to a substantial enhancement in the induction efficiency of ChP organoids. Moreover, the expression of early ChP developmental markers OTX2 and MSX1 was also detected in these organoids, suggesting progressive specification toward the ChP fate. Although these ChP organoids did not form fluid‐filled compartments, they could also secrete iCSF with proteomic and exosomal signatures closely resembling native CSF, establishing functional barrier properties, and exhibiting ventricular heterogeneity with predominant hindbrain identity. This niche‐mediated priming approach provides a robust, scalable platform for studying ChP development, disease modeling and therapeutic screening.

## 2 Results

### 2.1 Generation of Sporadic ChP Organoid‐Like Aggregates During Skin Organoids Induction

During the directed differentiation of hESCs (H9) into skin organoids [22], we observed the sporadic emergence of aggregates morphologically distinct from the typical skin organoids (Figure S1A). These aggregates exhibited a “tree‐like” structure, consisting of two distinct structural regions: one end resembling a “canopy” and the other a “trunk”. Comparative analysis with established organoid models revealed that these aggregates closely resembled ChP organoids [19]. Immunofluorescence staining confirmed that the skin organoids expressed skin lineage marker KRT15, contained typical hair follicle structures and abundant adipocytes, but barely expressed the ChP epithelial markers AQP1 and TTR (Figure S1B–E). In contrast, the aggregates strongly expressed AQP1 and TTR, while KRT15 level was very low. (Figure S1C–E). These findings indicated that the aggregates might be ChP organoids.

### 2.2 Switching hESCs Culture Medium From E8 to mTeSR1 Significantly Enhances the Induction Efficiency of ChP Organoids

**FIGURE 1:**
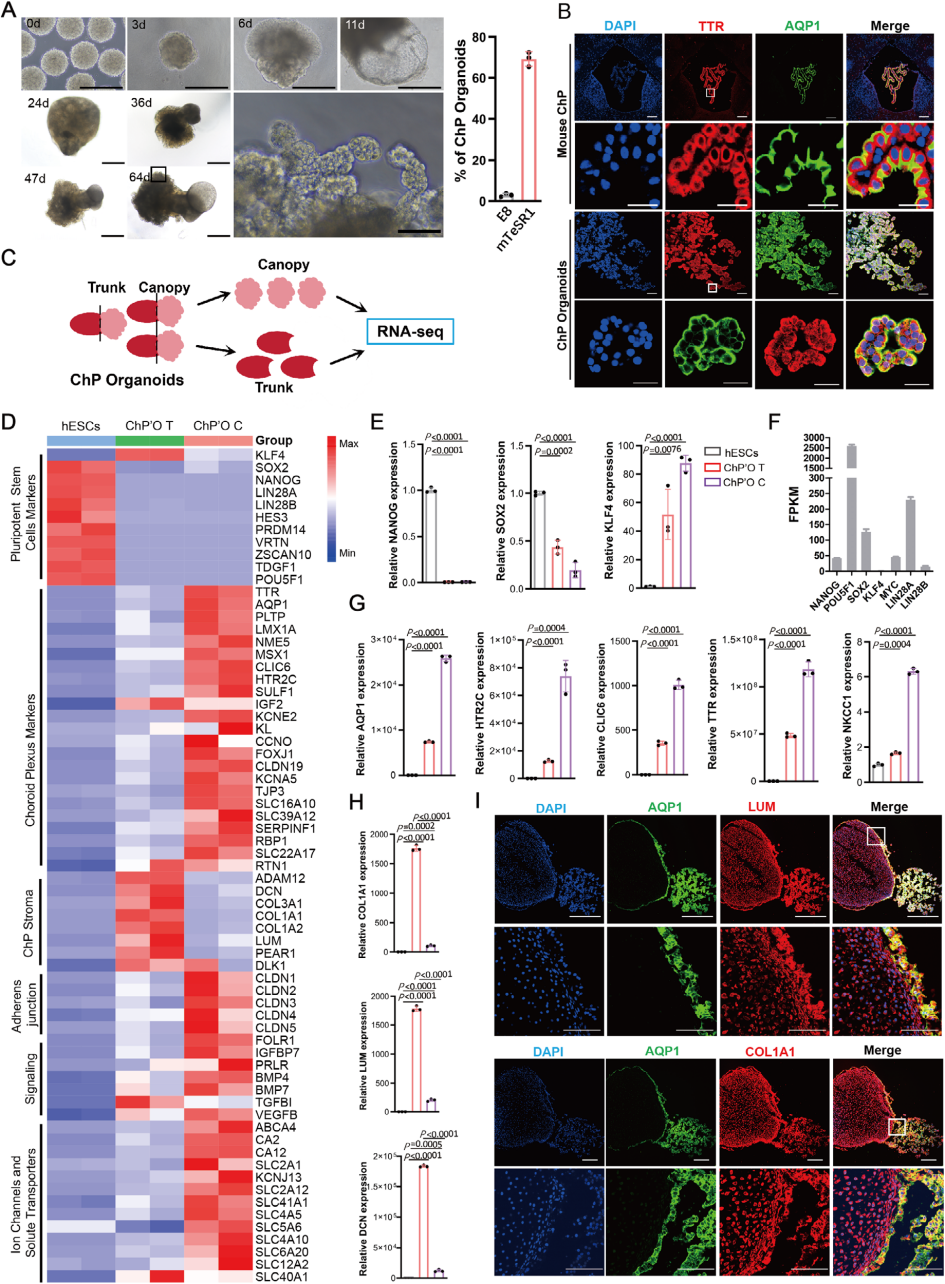
Switching hESC culture medium from E8 to mTeSR1 significantly enhances ChP organoids induction efficiency. (A) Morphologies of developing ChP organoids derived from hESCs (left). Graph shows quantification of induction efficiency of ChP organoids derived from hESCs cultured in E8 or mTeSR1 (right). Scale bars, 500 µm. Box scale bars, 100 µm. Statistical analysis was done by unpaired t‐test; data are presented as mean ± SD. n = 3. (B) Representative confocal images of fluorescent immunohistology for ChP epithelium markers TTR and AQP1 in ChP organoids at day 45 and in vivo ChP from an adult mouse. Cell nuclei were stained blue with 4’,6‐diamidino‐2‐phenylindole (DAPI). Scale bars, 100 µm (upper); 25 µm (lower). (C) Diagram describing the segmentation of aggregates and then applied for RNA‐seq. (D) Heatmap comparing the expression of hPSCs and ChP markers, and genes related to tight junction, cell signaling, ion channel, and solute transport genes within the bulk RNA transcriptomes of day 45 aggregates, and hESCs. Values are shown as Log2(TPM+1). (E) RT‐qPCR analysis shows the expression of the hPSCs markers *NANOG*, *SOX2* and *KLF4* in the ChP'O T, ChP'O C and hESCs. (F) Barplot showing the absolute expression, measured by RNA‐seq, of pluripotency markers in hESCs. FPKM, Fragments Per Kilobase of exon model per Million mapped fragments. Within the same sample, a higher FPKM value indicates a higher expression level. (G) RT‐qPCR analysis shows the expression of the ChP epithelium genes *TTR*, *AQP1*, *HTR2C*, *CLIC6* and *NKCC1* in hESCs, ChP'O T and ChP'O C. (H) RT‐qPCR analysis shows the expression of the ChP stroma markers *COL1A1*, *DCN* and *LUM* in hESCs, ChP'O T and ChP'O C. (I) Representative confocal images of fluorescent immunohistology for COL1A1, LUM and DAPI in ChP organoids at day 45. COL1A1 and LUM are shown in red, and AQP1 is shown in green. Cell nuclei are stained blue with DAPI. Scale bars, 500 µm. Box scale bars, 100 µm. Statistical analysis was done by unpaired t‐test; data are presented as mean ± SD.

The formation proportion of these aggregates during skin organoid induction was notably low (<3%), which would severely limit their applications in developmental biology and regenerative medicine. Therefore, improving their induction efficiency was urgently necessary. Both the nervous system and skin epithelium originate from the ectoderm [8, 13], and their developmental fate is determined by signaling pathways such as FGF, BMP, and WNT. Given the ChP epithelium develops along with the nervous system and is derived from neuroepithelial cells [2, 23], we hypothesized that priming PSCs toward a neural differentiation‐prone state might increase the formation rate of these aggregates. Previous studies have shown that mTeSR1 medium could promote neural differentiation of hESCs [20, 21]. Accordingly, we replaced E8 medium with mTeSR1 and assessed the induction efficiency after five passages. Surprisingly, the formation rate of these aggregates significantly increased from about 2.6% to approximately 68% (Figure 1A). The canopy region of these aggregates exhibited a distinct epithelial‐like structure, and displayed polarized apical staining for AQP1, resembling the ChP in the mouse ventricles (Figure 1A,B). Consistent with this, similar results could also be obtained using another hESCs cell line (H1), although the proportion was slightly lower (Figure S2A,B).

**FIGURE 2:**
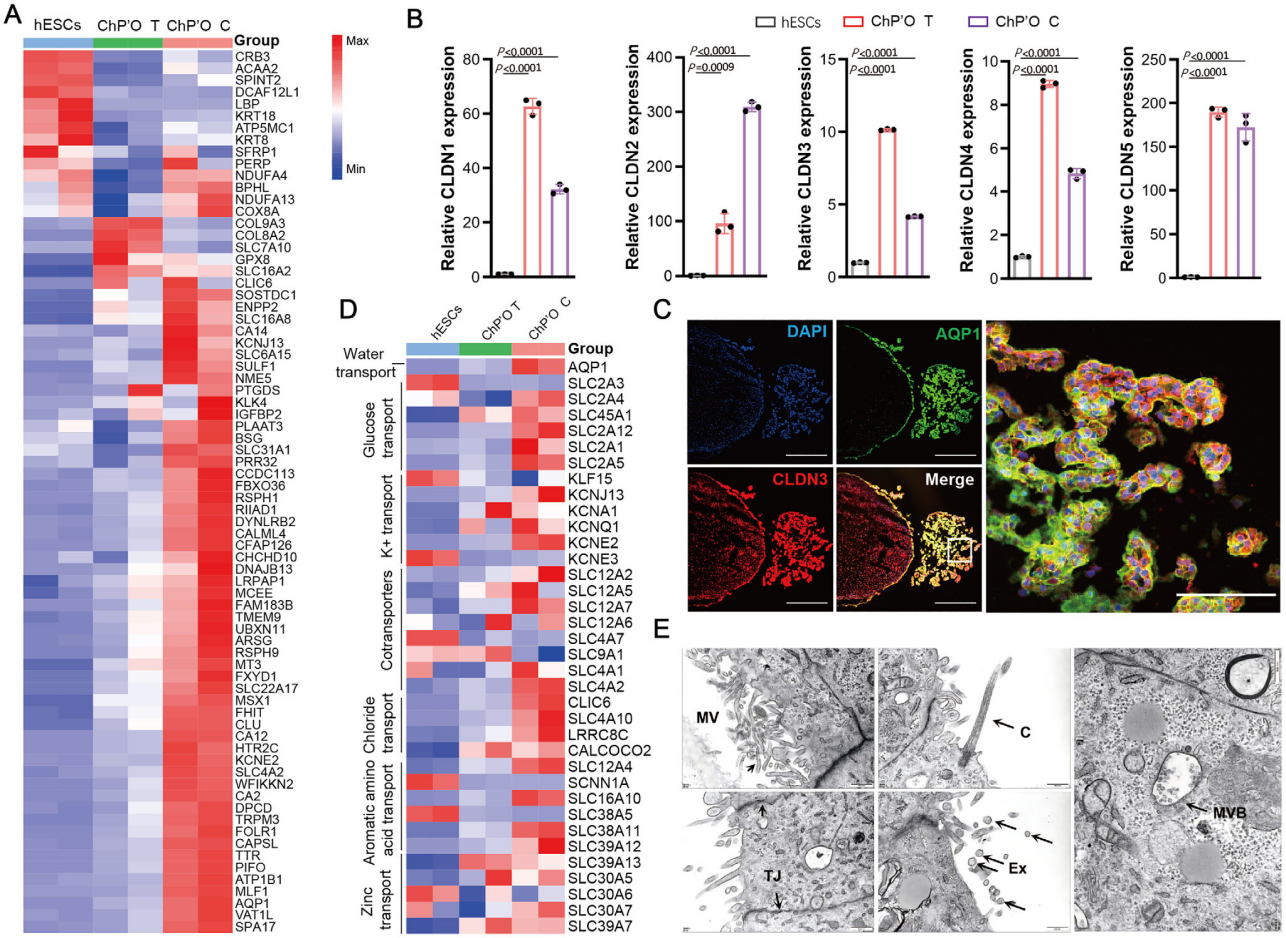
ChP organoids form a tight barrier. (A) Heatmap showing that 74 ChP epithelial genes identified in Dani et al., 2021 were enriched in these ChP organoids. (B) RT‐qPCR analysis shows the expression of the tight junction markers *CLDN1*, *CLDN2*, *CLDN3*, *CLDN4*, *CLDN5* in hESCs, ChP'O T and ChP'O C. (C) Representative confocal images of fluorescent immunohistology for AQP1 (green), CLDN3 (red) of 45 days ChP organoids. Cell nuclei are stained blue with DAPI. Right, high‐magnification image of the specimen in the left panel. Scale bars: 500 µm. Box scale bars, 100 µm. (D) Heatmap of normalized read counts of a curated set of genes encoding different classes of transporters expressed in hESCs and ChP organoids. (E) Electron micrographs of ChP organoids (day 45) showing microvilli (MV), cilia (C), tight junctions (TJ), extracellular vesicles (Ex), and multivesicular bodies (MVB). Scale bar:500 nm. Statistical analysis was done by unpaired t‐test; data are presented as mean ± SD.

To further investigate their identity, we performed RNA sequencing (RNA‐seq) on aggregates differentiated for 50 days, using hESCs as a control. Notably, to avoid overlooking rare but functionally important cell types, we did not pool all cells together from the entire aggregate for RNA‐seq. Instead, the aggregates were dissected at the junction between the “trunk” (ChP organoids’ trunk, ChP'O T) and the “canopy” (ChP organoids’ canopy, ChP'O C), and then the two portions were subjected to RNA‐seq separately (Figure 1C). Although this method might not achieve complete separation, it still allowed comparison of the two regions. Gene expression analysis confirmed that pluripotent markers such as SOX2 and NANOG were substantially downregulated in these aggregates compared to hESCs. In contrast, KLF4 expression was upregulated (Figure 1D). Real‐time quantitative PCR (RT‐qPCR) validated the RNA‐seq data (Figure 1E). This may be attributed to two factors: first, KLF4 expression is inherently low in conventional PSCs [24]. Indeed, analysis of RNA‐seq data showed that KLF4 had very low absolute expression in hESCs compared to other pluripotent factors (Figure 1F). Second, KLF4 is involved in regulating epithelial tissue development [25]. Regarding ChP epithelial markers, the expression of TTR, AQP1, CLIC6, HTR2C, and NKCC1 (SLC12A2) were markedly elevated, with higher levels in the canopy than in the trunk. Conversely, mesenchymal markers such as LUM, COL1A1, DCN, and DLK1 were more highly expressed in the trunk region (Figure 1D), which was validated by RT‐qPCR (Figure 1G,H). Immunofluorescence staining further demonstrated a strong expression of LUM and COL1A1 in the trunk, revealing their mesenchymal identity. Additionally, we observed that only the outermost layer of cells in the trunk expressed AQP1 (Figure 1I). As a result, the stroma portion was encapsulated by the epithelium, similar to the structure of the ChP tissue in the body. Recently, Dani et al. identified 86 genes highly expressed in mouse ChP using single‐cell RNA sequencing (scRNA‐seq) [26]. Among these, 74 genes (86.05%) were significantly enriched in these aggregates (Figure 2A), further supporting the high similarity between the aggregates and in vivo ChP.

A critical characteristic of ChP is barrier function [1]. Therefore, we first examined the expression of tight junction markers. Analysis of RNA‐seq data revealed substantial expression of tight junction proteins, such as CLDN1 and CLDN2 (Figure 1D). These findings were further confirmed by RT‐qPCR and immunofluorescence staining (Figure 2B,C). Moreover, genes associated with signal transduction, ciliogenesis, ion channels, and solute transporters were also significantly upregulated in these aggregates (Figure 2D and Figure S2C), consistent with the ciliated epithelial nature of the ChP. Electron microscopy further validated the presence of hallmark ultrastructural features of ChP epithelium, including abundant tight junctions, cilia, extensive microvilli, multi‐vesicular bodies, and numerous extracellular vesicles (Figure 2E). Taken together, the above results suggested that these tree‐like aggregates arising during skin organoid induction are ChP organoids, with the canopy corresponding to the epithelium and the trunk to the stroma of in vivo ChP.

### 2.3 ChP Organoids Secrete iCSF Resembling In Vivo CSF

**FIGURE 3:**
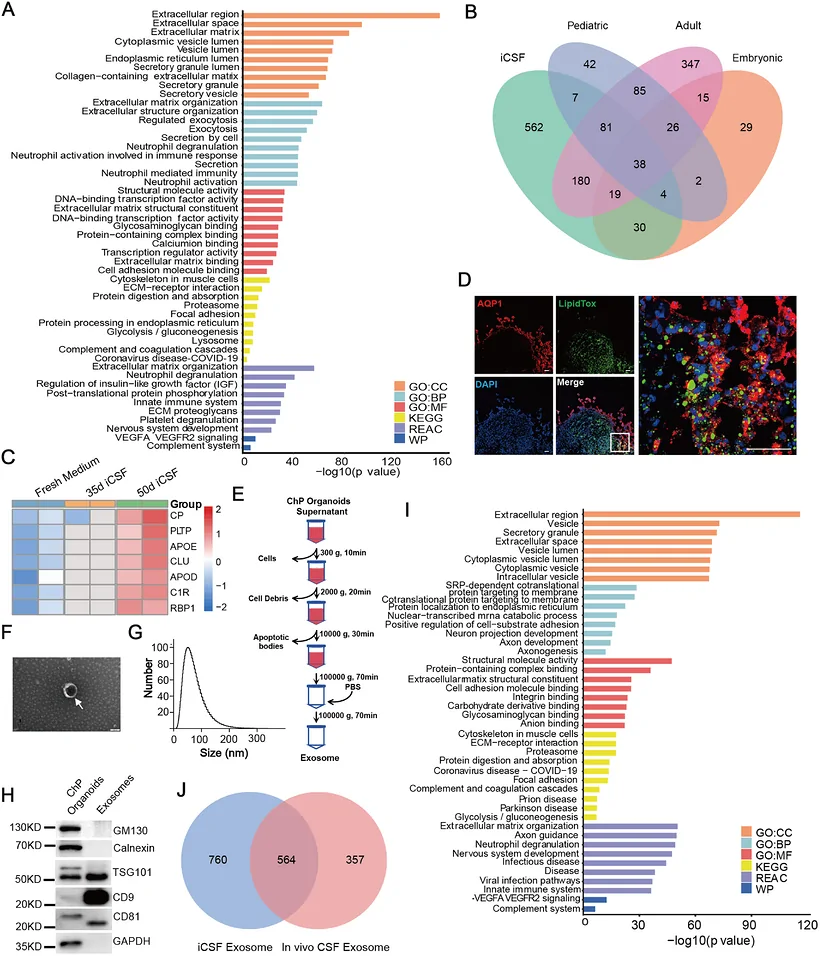
ChP organoids secrete iCSF resembling to in vivo CSF, and exosomes in iCSF are highly similar to in vivo CSF exosomes. (A) gProfileR analysis of proteins detected in 2 iCSF from 50 days ChP organoids samples, showing significant enrichments (p<0.05) for GO categories cellular component (GO:CC), molecular function (GO:MF), and biological process (GO:BP), KEGG, REAC, WP databases. Fresh medium was used as a control. (B) Venn diagram of proteins detected in 2 iCSF from 50 days ChP organoids samples. Overlap is shown for proteins detected in datasets of human adult CSF [29], pediatric CSF from healthy controls [31], and human embryonic CSF [30]. (C) Heatmap of protein levels for selected development and mature CSF components in ChP organoids iCSF over time. (D) Representative confocal images of ChP organoids at day 45 stained for lipid droplets (LipidTox) and AQP1. LipidTox is shown in green, and AQP1 is shown in red. Cell nuclei are stained blue with DAPI. Scale bar: 50 µm. Box scale bars, 100 µm. (E) Scheme of purification of exosomes from iCSF by differential centrifugation. (F) Representative electron micrographs of iCSF‐derived exosomes (white arrows). Scale bar: 100 nm. (G) Size distribution of exosomes in the CSF determined by Nanoparticle tracking analysis. (H) Immunoblotting for exosome markers CD9, CD81, and TSG101 in iCSF‐derived exosomes fractions and ChP organoids from the corresponding samples, The endoplasmic reticulum marker calnexin and the Golgi marker GM130 are used as negative controls. (I) gProfileR analysis of proteins detected in iCSF‐derived exosomes, showing significant enrichments (p<0.05) for GO categories cellular component (GO:CC), molecular function (GO:MF) and biological process (GO:BP), KEGG, REAC, WP databases. (J) Venn diagram showing overlap of proteins identified in iCSF exosomes and in vivo CSF exosomes from healthy controls [34].

The other crucial function of the ChP is the production and secretion of CSF, which is essential for brain development and homeostasis. Although these ChP organoids did not form a compartment structure, this does not abrogate their ability to secrete iCSF. Previous studies have shown that ChP explants cultured in vitro could produce CSF [27, 28]. Therefore, we collected conditioned medium (a mixture of iCSF and culture medium) from ChP organoids differentiated for 35 and 50 days, using fresh medium as a control, and performed mass spectrometry analysis. We detected 921 proteins in 50 days iCSF (Table S1). Gene Ontology (GO) analysis revealed enrichment in GO:CC categories “secretory granule lumen”, “extracellular region”, “extracellular space” and GO:BP categories “exocytosis”, “secretion by cell”, “neutrophil activation involved in immune response” (Figure 3A), consistent with known characteristics of CSF [18]. Furthermore, these proteins were also involved in biological processes such as “protein digestion and absorption”, “Proteasome”, “innate immune system” and “nervous system development”, aligning with established CSF functions (Figure 3A). Compared with published proteomic datasets of in vivo CSF [18, 29], we found that among the most abundant 100 proteins, 72 were detectable in vivo samples (Table S2). Moreover, proteins in iCSF highly overlapped with in vivo CSF proteins from various developmental stages (Figure 3B) [29, 30, 31], indicating gradual maturation of 50 days ChP organoids. Next, we examined proteins whose expression level would increase in postnatal and adult CSF. Indeed, we identified some lipoproteins, such as clusterin (CLU), APOE, APOD, and phospholipid transfer protein (PLTP), which increased over time (Figure 3C and Table S1). Similarly, we also observed a marked presence of lipid droplets in 50 days ChP organoids (Figure 3D). Additionally, we could detect many clinically relevant biomarkers in iCSF (Table S3), such as AHNAK [32].

Due to the absence of some cell types (e.g., endothelial cells, immune cells), ChP organoids could not produce certain proteins unique to in vivo CSF. To further ascertain the similarity to in vivo CSF, we examined proteins detectable in vivo but absent from iCSF. Reactome and WikiPathways analysis showed enrichment in “blood clotting cascade”, “platelet degranulation” and “Response to elevated platelet cytosolic Ca^2^^+^” (Figure S2D), indicating their blood origin. Thus, these proteins were not present in iCSF, as the ChP organoids lack vascular systems.

### 2.4 Exosomes in iCSF are Highly Similar to In Vivo CSF Exosomes

Compared with CSF, CSF exosomes contain abundant brain‐derived proteins, which can better reflect the expression of proteins in the ChP and the brain. Moreover, several known biomarkers for neurodegenerative diseases have been identified in CSF exosomes [2], indicating potential application value in disease diagnosis. In addition, some CSF exosomes contain bioactive substances (such as proteins, nucleic acids, and lipids), which can exert biological functions within and beyond the CNS.

In order to accurately determine the protein components and functions of iCSF exosomes, we first isolated exosomes from iCSF by using ultracentrifugation (Figure 3E). Transmission electron microscopy (TEM) analysis confirmed the presence of exosomes in the iCSF (Figure 3F), and the nanoparticle tracking analysis revealed the diameters of these exosomes were mainly concentrated between 60 and 120 nm (Figure 3G). These exosomes expressed canonical exosomal markers including CD9, CD81 and TSG101 (Figure 3H). Then, mass spectrometry was performed to characterize the protein components and functional properties of these exosomes. A total of 1324 proteins were identified (Table S4), 93 of which were among the top 100 proteins listed in the ExoCarta database (Table S5), such as CD9, Flot1/2 and Hspa8. ExoCarta is an online database platform that catalogs exosome‐specific data pertaining to proteins, RNAs and lipids (http://www.exocarta.org) [33]. GO analysis revealed enrichment in GO:CC categories “extracellular region”, “lumen” and “vesicle”, while GO:BP categories “neuron projection development”, “axon development” and “axonogenesis”, indicating strong association with exosomal biology and neural system development. The KEGG pathway analysis further demonstrated enrichment not only in biological processes closely related to CSF functions such as “ECM‐receptor interaction”, “Protein digestion and absorption” and “Proteasome”, but also in subcategories linked to neurological disorders, including “Parkinson's disease”. Finally, Reactome pathway analysis highlighted significant enrichment in a series of signaling pathways and biological processes related to the functions of in vivo CSF exosomes, including “Axon guidance”, “Neutrophil degranulation”, “Nervous system development”, “Infectious disease” and “Innate Immune System” (Figure 3I). More importantly, compared with published proteomic data [34] of in vivo CSF exosomes revealed a large overlap between them (Figure 3J). In addition, proteins in iCSF exosomes highly overlapped with iCSF proteins (Figure S2E). Collectively, these results demonstrate that exosomes present in iCSF closely resemble those found in in vivo CSF.

### 2.5 ChP Organoids may Represent a Mixture of ChP From Different Ventricles and are Susceptible to SARS‐CoV‐2 Infection

The ChP is a highly vascularized tissue that is located within each ventricle of the brain: the lateral/telencephalic ventricle (1 V ChP), third/diencephalic ventricle (3 V ChP), and fourth/hindbrain ventricle (4 V ChP). Previous studies have identified region‐specific markers, with 1 V ChP highly expressing LY6E, EMX2 and SIX3 [35], 3 V ChP enriched for FHIT and FAM166B [26], and 4 V ChP specifically expressing PENK, SOD3 and WNT5A [35, 36]. Analysis of our RNA‐seq data confirmed the expression of these markers in ChP organoids (Figure S3A), which was further validated by RT‐qPCR (Figure S3B). However, we observed relatively low absolute expression of the 3 V ChP markers FHIT and FAM166B, while markers for 1 V ChP (LY6E, EMX2, SIX3) and 4 V ChP (PENK, SOD3, WNT5A) were expressed at higher levels (Figure S3C). In addition, PENK and SOD3 proteins were detected in both iCSF and its exosomes. Moreover, we also observed WNT5A in the exosomes, although its abundance was very low (Table S4). These findings suggest that the ChP organoids may represent a mixture of ChP epithelium from different ventricles, with 4 V ChP being most abundant, followed by 1 V ChP, and 3 V ChP the least. This differs from previously reported telencephalic ChP organoids [18], which were derived based on telencephalic organoid protocol.

Studies have shown that ChP epithelium was susceptible to SARS‐CoV‐2 infection [19, 37, 38]. We first examined the expression of SARS‐CoV‐2 receptor proteins ACE2, NRP1 and TMPRSS2 in these ChP organoids. Compared to hESCs, ACE2 and NRP1 levels were substantially upregulated in ChP organoids, with ACE2 showing a more pronounced increase—consistent with a previous report [19]. In contrast, TMPRSS2 expression was higher in hESCs (Figure S4A), which was confirmed by RT‐qPCR (Figure S4B). Study on human eggs, zygotes, and embryos indicated that TMPRSS2 was expressed in these samples, but with the highest abundance in blastocysts [39]. Since embryonic stem cells (ESCs) are derived from the inner cell mass of blastocysts, the higher TMPRSS2 level in hESCs is understandable.

We next evaluated whether ChP organoids were susceptible to SARS‐CoV‐2 infection. To facilitate comparison, we first generated brain organoids. Morphologically, there were significant differences between brain organoids and ChP organoids (Figure S4C). The former could form distinct neural tube‐like structures, highly expressing neural cell markers βIII Tubulin and neural stem cell marker SOX2, but lacking ChP markers TTR and AQP1. In contrast, ChP organoids exhibited epithelial‐like organization, expressed lower levels of βIII‐Tubulin, and were nearly negative for SOX2 (Figure S4C–E). RT‐qPCR further confirmed that brain organoids highly expressed neuronal markers DCX and FOXG1, whereas ChP organoids showed higher expression of ChP epithelial markers TTR, AQP1, and HTR2C (Figure S4F). Importantly, the expression level of ACE2 was significantly higher in ChP organoids (Figure S4G). We then infected both organoid types with SARS‐CoV‐2 spike pseudovirus and observed a markedly higher infection rate in ChP organoids (Figure S4H), aligning with previous findings [19]. Consistent with this, the KEGG analysis of iCSF and exosomal proteins also showed that they were significantly enriched in “coronavirus disease‐COVID‐19” (Figure 3A,H). Together, these results indicate that ChP organoids, like their in vivo counterparts, are susceptible to SARS‐CoV‐2 infection, supporting their utility as an in vitro ChP model for studying viral infection.

### 2.6 Switching From E8 to mTeSR1 Medium Enhances the Neural Priming of hESCs

**FIGURE 4:**
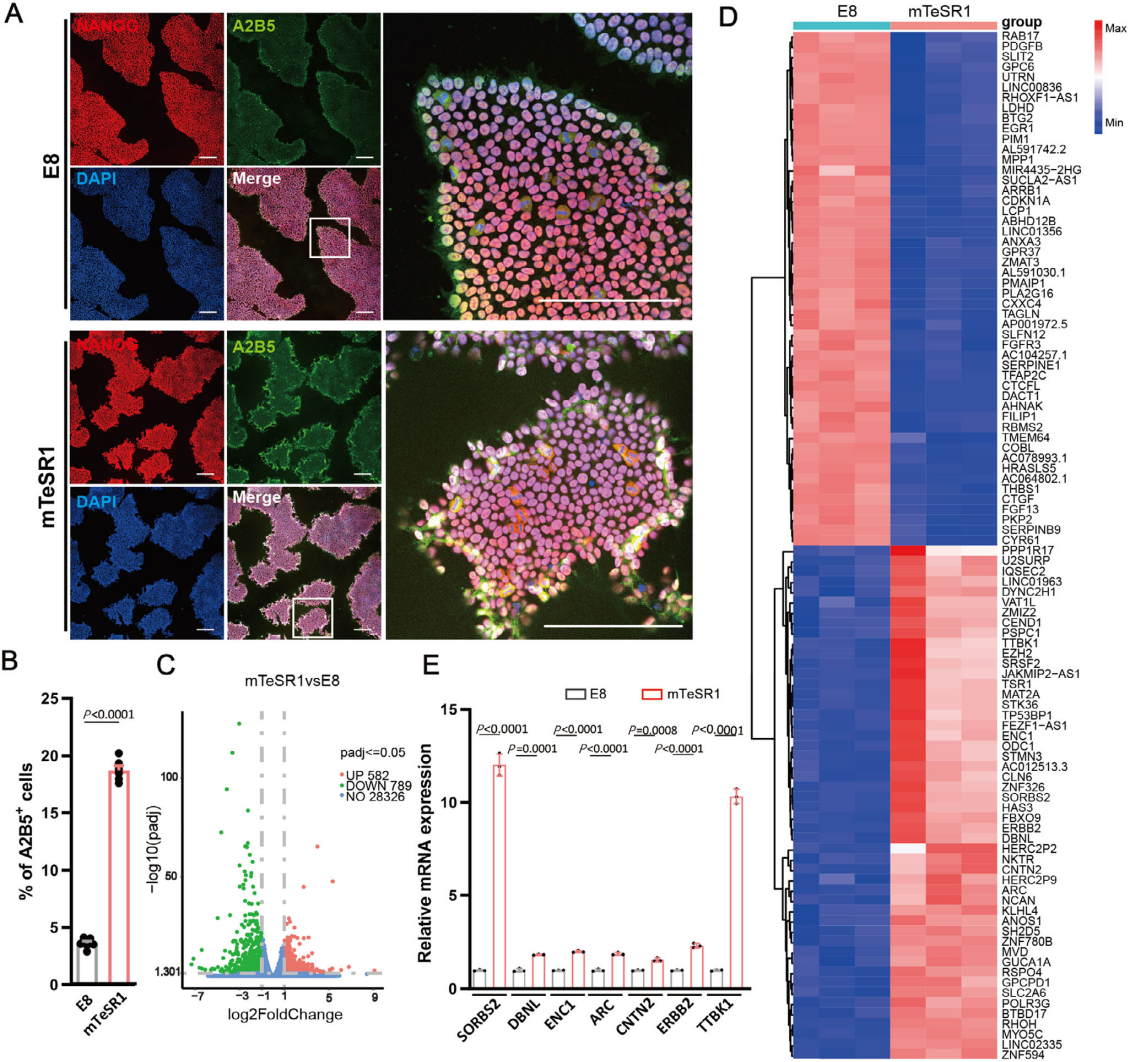
Neural lineage‐specific markers can predict subsequent in vitro differentiation of hESCs. (A) Expression level of the predictive neural progenitor cell marker A2B5 was greater in mTeSR1‐cultured hESCs. Scale bar: 200 µm. (B) Quantification of the A2B5 in the immunofluorescent staining shown in (A). (C) Volcano plot summarizing differential gene expression data obtained from RNA‐seq analysis between E8 and mTeSR1‐cultured hESCs. Upregulated genes with log2‐Fold Change (mTeSR1/E8) > 1 and Padj‐value < 0.05 are marked in red, while downregulated genes with log2‐Fold Change (mTeSR1/E8) < ‐1 and Padjvalue < 0.05 are marked in green. Padj is adjusted value calculated by Wald test statistic corrected for multiple testing. (D) Heat map showing the differentially expressed top 50 genes between E8 and mTeSR1‐cultured hESCs. (E) RT‐qPCR analysis of the expression of some upregulated genes shown in (D). Statistical analysis was done by unpaired t‐test; data are presented as mean ± SD.

Self‐renewal and differentiation are two fundamental properties of hESCs, and the expansion medium has been shown to influence these behaviors. For example, compared to conventional mouse embryonic fibroblast‐conditioned medium (MEF‐CM), mTeSR1 medium promotes a stronger neural lineage differentiation propensity in hESCs [20, 21]. Moreover, Jung Bok Lee et al. demonstrated that mTeSR1 increased the neural differentiation priming of hESCs, which correlated with elevated expression of the neural precursor marker A2B5 [21]. Since skin organoid induction was originally performed using hESCs maintained in E8 medium [22], we asked whether replacing E8 with mTeSR1 would enhance their neural priming. After switching hESCs medium from E8 to mTeSR1 for five passages, we found that pluripotent markers SOX2, NANOG, and KLF4 remained unaffected (Figure S5A,B). However, the expression level of A2B5 significantly increased, and it was mainly located in the peripheral cells of the hESCs clones (Figure 4A,B), consistent with a previous report [40], suggesting that mTeSR1 medium might enhance the neural priming of hESCs.

**FIGURE 5:**
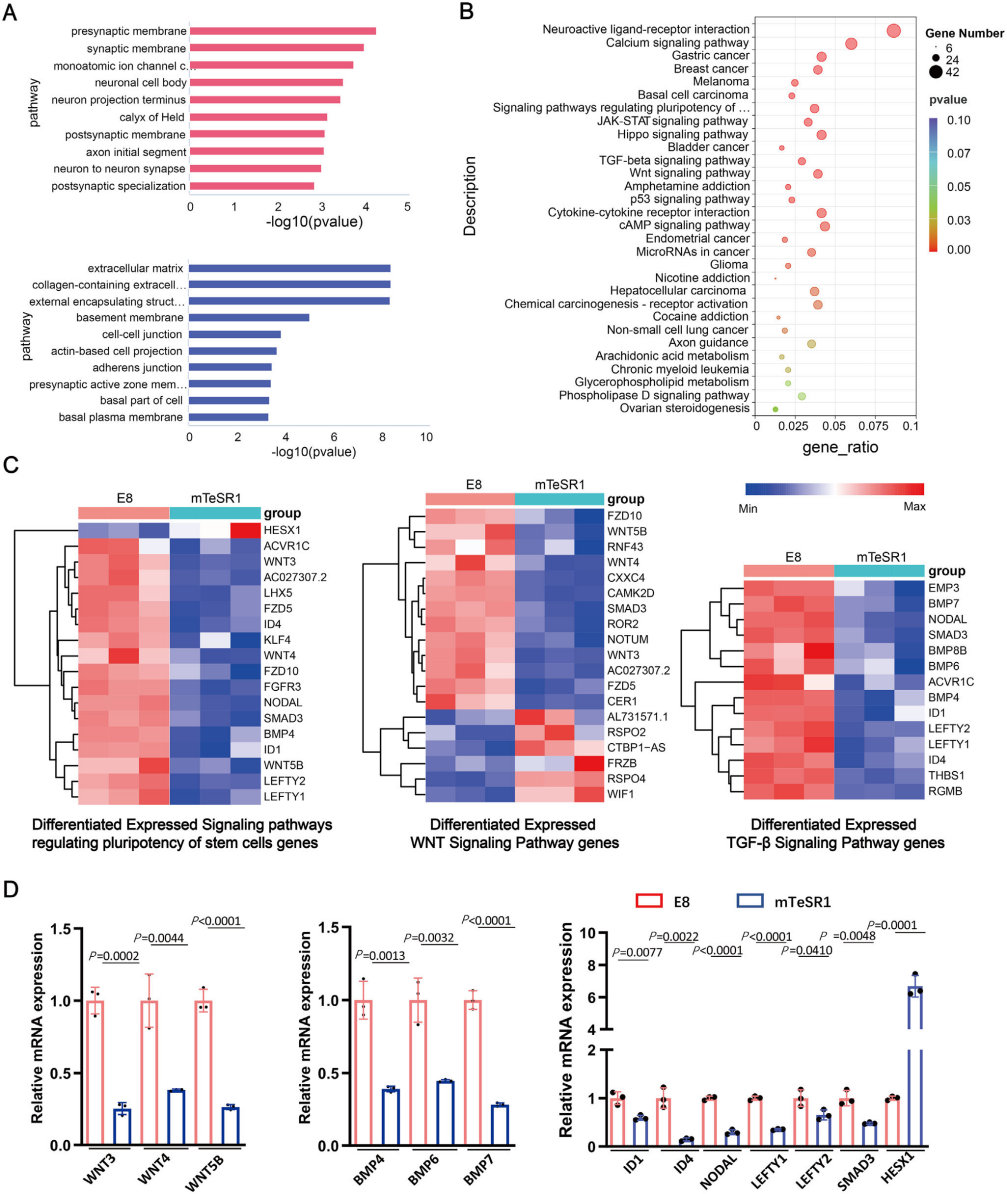
Switching from E8 to mTeSR1 medium enhances the neural priming of hESCs. (A) GO analysis of the functional annotations of differentially expressed genes, including upregulated genes (top) and downregulated genes (bottom). (B) Top 30 enriched pathways identified in hESCs when switching from E8 to mTeSR1. (C) Altered expression of functionally related genes of WNT, TGF‐β and regulating pluripotency of stem cell genes pathways detected by RNA‐seq analysis of mTeSR1 culture. (D) RT‐qPCR analysis of the expression of some regulated genes shown in (C). Statistical analysis was done by unpaired t‐test; data are presented as mean ± SD.

To elucidate the mechanism by which mTeSR1 enhanced neural priming, we then performed RNA‐seq on hESCs cultured under E8 and mTeSR1. Relative to E8, mTeSR1 culture led to upregulation of 582 genes and downregulation of 789 genes (Figure 4C). Figure 4D showed the most significantly differential genes, including 50 upregulated and 50 downregulated genes. Many upregulated genes were involved in neural differentiation and nervous system development, whereas downregulated genes were associated with extracellular matrix and cell‐cell adhesion. Seven representative upregulated genes were selected for RT‐qPCR validation. All of the genes were upregulated in mTeSR1, consistent with the RNA‐seq (Figure 4E). For instance, SORBS2 encodes ArgBP2 (Arg/c‐Abl kinase‐binding protein 2) in non‐neural tissues, while in the brain its splice variant nArgBP2 is highly expressed in the cortex, amygdala and hippocampus, playing important roles in dendritic development and excitatory synaptic transmission [41]. NRG1/ErbB2 signaling plays an essential role in nervous system development, such as neuronal guidance, neural differentiation, and the formation of the neuromuscular junction [42]. As expected, the protein levels of SORBS2 and ERBB2 also increased in hESCs cultured in mTeSR1 (Figure S5C). GO analysis indicated that the upregulated genes were enriched in terms such as “postsynaptic membrane”, “synaptic membrane”, “neuronal cell body” and “neuron projection terminus” (Figure 5A). Gene cluster analysis also confirmed that most genes associated with these processes were upregulated (Figure S5D). In contrast, downregulated genes were related to “extracellular matrix”, “basement membrane” and “external encapsulating structure” (Figure 5A). This indicated that mTeSR1 drove hESCs toward neural lineage commitment, consistent with previous studies [20, 21]. Furthermore, KEGG pathway analysis also revealed that many categories were related to neurogenesis, including “neuroactive ligand‐receptor interaction”, “Calcium signaling pathway”, “Cytokine‐cytokine receptor interaction” and “Axon guidance” (Figure 5B). Among the top 20 altered pathways, “the neuroactive ligand‐receptor interaction pathway” was the most significantly upregulated (Figure S5E). This pathway plays crucial roles in nervous system development by regulating gene expression and cellular signaling [43]. Additionally, we observed significant changes in pathways closely associated with pluripotency and embryonic development, such as “Wnt signaling pathway”, “TGF‐β signaling pathway” and “Signaling pathways regulating pluripotency of stem cells” (Figure 5B). In‐depth analysis of the genes showed downregulation of key genes in these pathways (Figure 5C,D). Moreover, we examined the protein levels of β‐catenin, a core component of the Wnt signaling pathway [44], and BMP4, a key protein in the TGF‐β pathway, and found that their expression levels indeed decreased (Figure S5C). Studies have indicated that suppression of Wnt and TGF‐β signaling promotes neural commitment of hESCs [8], and reduced expression of ID1, ID4, NODAL, SMAD3, LEFTY1 and LEFTY2 also favors neural differentiation [45]. Conversely, HESX1—a gene within the pluripotency regulatory network—was upregulated (Figure 5C,D). HESX1 is an important regulator of neural development, and its knockdown in hESCs impairs neural differentiation [46]. Thus, elevated HESX1 expression may further enhance the neural differentiation potential of hESCs. In summary, these results demonstrate that switching from E8 to mTeSR1 significantly enhances the neural priming of hESCs.

### 2.7 mTeSR1 Enhances Neuroepithelial Cells Differentiation of hESCs, Improving ChP Organoid Induction Efficiency

**FIGURE 6:**
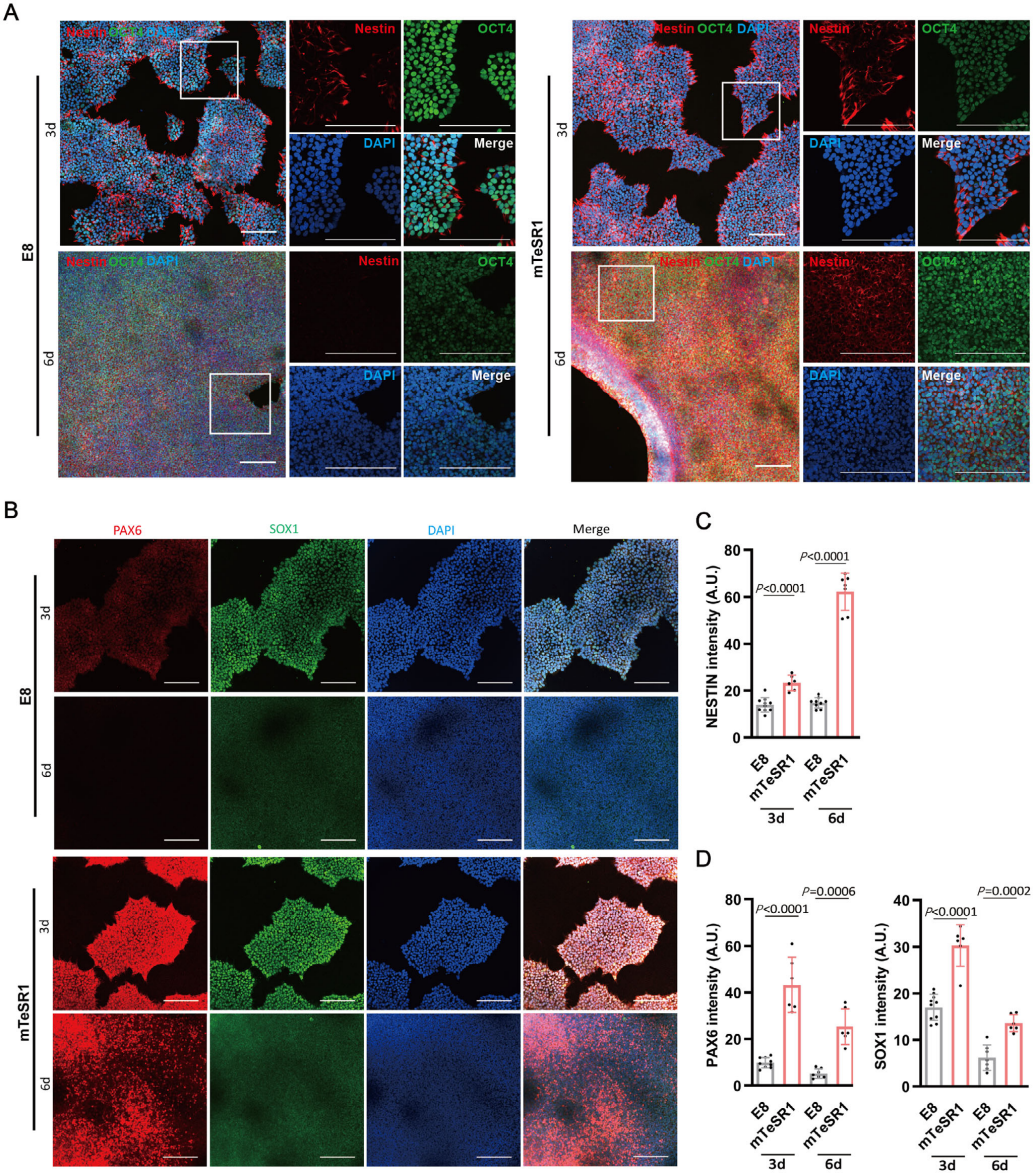
Switching from E8 to mTeSR1 medium enhances the directed differentiation of neuroepithelia cells from hESCs. (A,B) Representative confocal images of fluorescent immunohistology for neuroepithelia cells markers NESTIN, PAX6, and SOX1 after directly differentiating for 3 days and 6 days for E8 and mTeSR1 cultured hESCs respectively. Cell nuclei were stained blue with DAPI, and the SOX1 is shown in green, while NESTIN and PAX6 are shown in red. Scale bar: 200 µm. (C,D) The statistical analysis of the mean fluorescence intensity of NESTIN, PAX6 and SOX1 shown in (A,B). Statistical analysis was done by unpaired t‐test; data are presented as mean ± SD; n≥6 images.

To validate that mTeSR1 promotes neural differentiation of hESCs, we directed hESCs toward neural lineage induction in vitro [21]. Given that the ChP epithelium is derived from neuroepithelial cells, we examined the expression of neuroepithelial cell markers SOX1, PAX6, and NESTIN. After 3 days, differentiated cells originated from hESCs under both media (E8 and mTeSR1) could express these markers (Figure 6A,B). However, compared to E8‐cultured cells, the proportion and intensity of these markers were higher in mTeSR1‐cultured cells (Figure 6A–D). By day 6, expression levels of SOX1 and PAX6 declined in both conditions, but the proportion and intensity remained also significantly higher in mTeSR1‐cultured cells (Figure 6D). Conversely, NESTIN expression increased in mTeSR1‐cultured cells (Figure 6A,C). These results indicated that mTeSR1 enhanced the neuroepithelial differentiation propensity of hESCs. Considering this was observed under directed differentiation, we then asked whether a similar phenomenon occurred during skin organoid induction. Unexpectedly, after dissociating hESCs colonies into single cells, we found that hESCs maintained in E8 failed to form aggregates using the hanging drop method, while mTeSR1‐cultured hESCs aggregated normally. If these single cells were forced to aggregate at the bottom of ultralow‐adherent U‐bottom 96‐well plates by centrifugation, there was no difference between the two media (Figure S6A). Previous study suggested that human induced pluripotent stem cells (hiPSCs) cultured in mTeSR1 secreted more growth‐regulatory factors, while those in E8 produced more survival factors and extracellular proteins [47]. By analyzing RNA‐seq data from undifferentiated hESCs under both conditions, we also observed a similar phenomenon. GO analysis showed higher activity in “collagen binding”, “extracellular matrix”, “cytokine activity” and “growth factor activity” in E8‐cultured cells (Figure S6B). This suggested that E8‐cultured hESCs may produce less extracellular matrix on their surface, hindering gravity‐dependent aggregation in hanging drops‐a limitation overcome by centrifugation. Despite differences in aggregation efficiency, the resulting aggregates showed no difference in the expression of pluripotent markers of hESCs under both conditions (Figure S6C).

**FIGURE 7:**
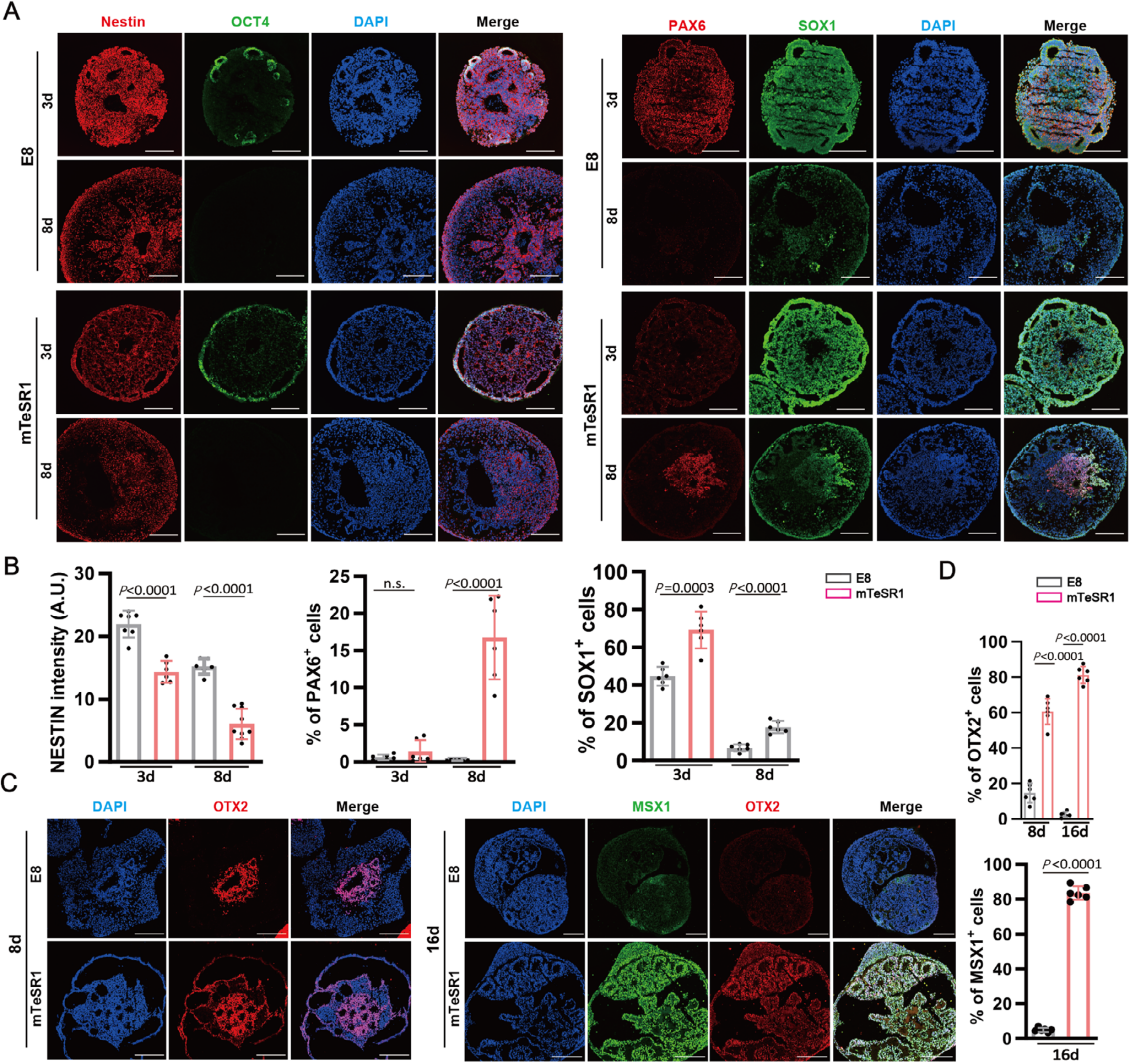
mTeSR1 enhances neuroepithelial cells differentiation of hESCs and promotes early ChP lineage specification during Skin organoid induction. (A) Representative confocal images of fluorescent immunohistology for neuroepithelia cells markers NESTIN, PAX6, and SOX1 after differentiating for 3 days and 8 days for E8 and mTeSR1 cultured hESCs during skin organoids induction, respectively. Cell nuclei were stained blue with DAPI, and the SOX1 is shown in green, while NESTIN and PAX6 are shown in red. Scale bars, 250 µm. (B) Statistical analysis of the mean fluorescence intensity of NESTIN, PAX6, and SOX1 shown in (A). (C) Representative confocal images of fluorescent immunohistology for genes (OTX2 and MSX1) involved in ChP development after differentiating for 8 days and 16 days for E8 and mTeSR1 cultured hESCs during skin organoids induction, respectively. Cell nuclei were stained blue with DAPI, and the MSX1 is shown in green, while OTX2 is shown in red. Scale bars, 250 µm. (D) Statistical analysis of the mean fluorescence intensity of OTX2 and MSX1 shown in (C). Statistical analysis was done by unpaired t‐test; data are presented as mean ± SD; n≥6 images.

Similar to the directed differentiation, after 3 days in skin organoid induction medium, OCT4 expression rapidly decreased, while the expression of neuroepithelial markers SOX1, PAX6, and NESTIN increased under both conditions (Figure 7A). By day 8, the expression level of these markers gradually decreased in E8‐cultured cells. However, PAX6, a protein typically expressed in the nucleus, was almost exclusively expressed in the cytoplasm (Figure 7A,B), and the reason remains to be investigated. In contrast, under mTeSR1 condition, NESTIN and PAX6 levels were relatively low on day 3, while SOX1 remained high. By day 8, NESTIN and SOX1 decreased, but PAX6 expression increased and was notably localized to the nucleus, co‐localized with SOX1 (Figure 7A,B). These results indicated that the neuroepithelial differentiation potential of hESCs cultured under mTeSR1 has also increased during the skin organoid induction. Following their differentiation from embryonic stem cells into neuroepithelial cells, the latter subsequently give rise to choroid plexus epithelium. Previous studies have shown that OTX2 and MSX1 are expressed in developing or immature ChP cells [18], and OTX2 also plays important roles in brain development [48]. So we examined OTX2 expression in early ChP organoids and found that at day 8, OTX2 expression was significantly higher in organoids derived from mTeSR1‐cultured hESCs, and this proportion further increased by day 16. In contrast, organoids derived from E8‐cultured hESCs showed much lower OTX2 expression, with a substantial decrease observed by day 16 (Figure 7C,D). Furthermore, we also observed a significantly higher proportion of MSX1 expression in mTeSR1‐derived organoids at day 16 (Figure 7C,D). Collectively, these results demonstrated that the enhanced neural differentiation propensity of hESCs conferred by mTeSR1 culture was gradually directed toward a Chp fate under skin organoids induction protocol.

As both the ChP and the skin originate from the ectoderm, and the ChP epithelium differentiates from neuroepithelial cells, this can explain why switching the hESCs culture medium from E8 to mTeSR1 significantly increases the formation efficiency of ChP organoids during the induction of skin organoids. In summary, these results demonstrate that the mTeSR1 medium enhances the neural differentiation priming of hESCs, thereby substantially improving their ability to generate ChP organoids in the skin organoid induction culture system.

## 3 Discussion

Due to their unlimited proliferative capacity and multilineage differentiation potential, hPSCs hold great promise in developmental biology and regenerative medicine [49, 50]. Recent advances in organoid technology have provided powerful tools for realizing these applications [51]. When directing PSCs toward organoids formation in vitro, researchers often rely on precisely activating or inhibiting certain signaling pathways by combining different small molecule compounds and proteins, but the influence of the culture microenvironment (niche) on subsequent lineage commitment is frequently overlooked. As we all know, the niche plays a critical role in regulating PSCs self‐renewal and differentiation [52, 53]. However, the diversity of hESCs culture systems creates different niches that influence hESCs fate. By engineering these niche properties, the rate and differentiation pathway of hESCs can be controlled [54]. For instance, hESCs maintained in mTeSR1 exhibit higher proliferative potential and a stronger neural bias compared to those cultured in MEF‐CM [20, 21]. Therefore, in addition to developing the medium for inducing hPSCs toward specific lineages, selecting an appropriate expansion medium is also a crucial prerequisite for successful clinical translation.

During skin organoid induction, we observed the sporadic formation of ChP organoids. Considering that the ChP shares an ectodermal origin with the skin, the occasional emergence of ChP organoids during the skin organoids directed differentiation is understandable. Previous studies demonstrated that BMP4 (5 ng/ml) is sufficient to induce ChP epithelial fate from hESCs‐derived neuroepithelial progenitors. Within the first three days of hESCs differentiating into skin organoids, the concentration of BMP4 was 2.5 ng/ml. Until the 12th day, BMP4 remained present in the differentiation medium, although its concentration gradually decreased. Moreover, a subset of cells indeed express the neuroepithelial cell markers SOX1, NESTIN and PAX6 during early differentiation. This may explain why a very small proportion of ChP organoids arise during skin organoid induction. Additionally, aggregates treated with BMP inhibitor (LDN189193) and high concentration basic fibroblast growth factor (bFGF) could give rise to cranial neural crest (CNC) cells [22], which can further differentiate into various mesenchymal lineages, including ChP stroma [26, 55]. This may account for the relatively abundant mesenchymal cell population observed within these ChP organoids.

The ChP epithelium differentiates from neuroepithelial cells [2, 23]. Therefore, enhancing the neural priming of hESCs—particularly their neuroepithelial differentiation propensity—could improve the induction efficiency of ChP organoids. Previous studies indicated that hESCs cultured in mTeSR1 exhibited a stronger bias toward neural lineage commitment [20, 21]. Accordingly, switching the hESCs medium from E8 to mTeSR1 led to a remarkable increase in ChP organoids formation efficiency. This may be attributed to two reasons: first, mTeSR1 culture significantly activates neural related pathways, such as “the neuroactive ligand‐receptor interaction pathway”, thereby strengthening neural predisposition. Second, downregulation of the Wnt and TGF‐β signaling pathways may further promote neural commitment in hESCs. Thus, the combined effects of mTeSR1‐enhanced neural priming and BMP4‐driven ChP epithelial specification likely synergistically boost the efficiency of hESCs differentiation toward ChP lineages.

It is well known that, given the high complexity of the brain and the diversity of culture conditions for PSCs, a wide variety of brain organoid models have been generated to date [56]. Since mTeSR1 culture enhances the neural differentiation potential of hESCs, it raises the question of whether this approach could improve the induction efficiency of certain brain organoid subtypes. Furthermore, for organoid induction protocols that do not routinely use mTeSR1 for PSCs expansion, what changes might occur if the cells were cultured in mTeSR1? Moreover, is it possible to rationally design the in vitro culture microenvironment of PSCs according to the developmental requirements of specific tissue organoids, thereby enhancing their differentiation efficiency?

**FIGURE 8:**
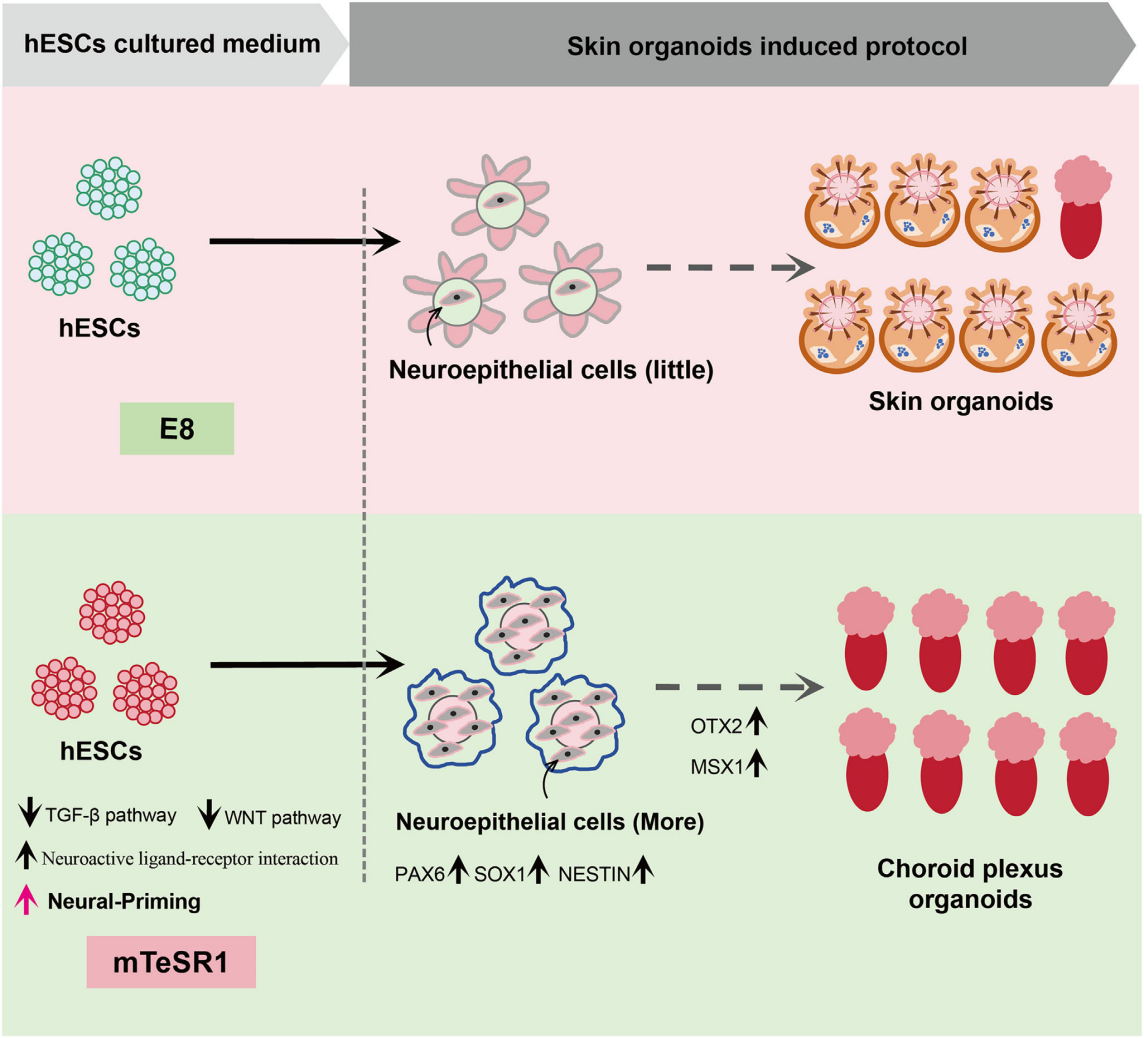
Schematic illustration of mTeSR1 culture‐enhanced neural differentiation propensity promoting ChP organoid formation under skin organoid induction condition. Compared to E8, mTeSR1 culture downregulated WNT and TGF‐β signaling, and upregulated neural differentiation pathways, enhancing neural priming. Under skin organoid induction conditions, mTeSR1‐cultured hESCs generated organoids with significantly higher neuroepithelial cells differentiation. With the progressive expression of early ChP developmental genes, including OTX2 and MSX1, the organoids were gradually directed toward a ChP fate.

In summary, we found that switching hESCs medium from E8 to mTeSR1 can significantly enhance their neural priming, thereby increasing the proportion of neuroepithelial cells differentiation. This ultimately leads to a substantial improvement in the induction efficiency of ChP organoids (Figure 8). These ChP organoids provide a robust tool for studying ChP development and regenerative medicine. Furthermore, the capacity to secrete iCSF also makes them suitable for disease modeling, such as hydrocephalus [57], monitoring disease‐related biomarker secretion and drug screening.

## 4 Advantages and Limitations of the Study

Although the ChP is an essential component of the brain, it has received less attention compared to other brain structures, and only a few ChP organoid models have been established. Morphologically, our ChP organoids resemble those established by Jacob et al. [19], including clustered epithelial structures. However, there is also a notable difference: the latter contain little to no mesenchymal cells. Moreover, a clear boundary exists between the epithelial and mesenchymal compartments. Interestingly, the mesenchymal core is enveloped by an epithelial layer, making these structures more reminiscent of the in vivo ChP. This architecture may offer advantages for studying stroma‐epithelium interactions.

A key function of the ChP is to secrete CSF and establish the BCSFB, which prevents free passage of molecules from the systemic circulation into the CSF, thereby maintaining brain homeostasis. ChP organoids reported by Pellegrini et al. formed fluid‐filled compartments and exhibited BCSFB‐like selective permeability, making them suitable for drug screening [18]. Although our ChP organoids do not form similar enclosed cavities, they can also secrete iCSF, which contains the specific proteins of CSF and many known disease biomarkers (e.g., clusterin [CLU] and Apolipoprotein E [APOE]). In this regard, these ChP organoids resemble in vitro cultured ChP explants. In addition, lacking compartment structure may offer certain advantages. For instance, due to the spatial constraints of the compartments, the production of iCSF by ChP organoids is extremely limited. Moreover, their cavities are prone to rupture during culture, and CSF collection typically requires disruption of the structure, allowing only a single harvest. In contrast, our ChP organoids secrete iCSF without spatial confinement, enabling repeated, non‐destructive collection—a feature that may be advantageous for CSF and exosomes research as well as therapeutic applications. Of course, the absence of a sealed cavity also limits their utility in permeability‐based compound screening. Additionally, exosomes isolated from iCSF contain proteins highly similar to those found in in vivo CSF exosomes. Importantly, there is substantial overlap between exosomal proteins and iCSF proteins, including many clinically relevant biomarkers (Table S3), suggesting that iCSF‐derived exosomes may, to some extent, perform or even substitute for iCSF, with potential implications for disease treatment.

Additionally, although we have elucidated why hESCs cultured in mTeSR1 exhibit a higher neural differentiation propensity, it remains unclear how the increased neuroepithelial cells are directed toward a Chp epithelial fate. While we observed that organoids derived from mTeSR1‐cultured hESCs express higher levels of the early ChP markers OTX2 and MSX1, our analysis was limited to only two time points and lacks a systematic investigation covering the entire developmental time course. Furthermore, numerous genes are involved in ChP development. The precise molecular mechanisms—including potential cross‐regulatory networks and the possible involvement of additional factors—remain to be fully elucidated in future studies.

## 5 Experimental Methods

### 5.1 Ethics Statement

The mouse study has been approved by the Animal Care and Use Committee of Guizhou Medical University (2602033). Human ESCs (hESCs) experiments were performed at the Center of Tissue Engineering and Stem Cells of Guizhou Medical University and followed the 2016 and 2021 Guidelines released by the International Society for Stem Cell Research (ISSCR). hESCs work was reviewed and approved by the Ethics Committee of Guizhou Medical University.

### 5.2 Cell Lines

The H9 hESCs were provided by Dr. Tianqing Li from Kunming University of Science and Technology, and the H1 hESCs were provided by Dr. Ping Zheng from Kunming Institute of Zoology, CAS. All cell culture experiments involving hESCs were approved by the Stem Cell Oversight Committee at the Guizhou Medical University and carried out in accordance with the approved guidelines.

### 5.3 HESCs Culture

The cell lines tested negative for mycoplasma prior to experimentation. hESCs were maintained on 35 mm cell culture dishes (Jet Biofil, TCD010035) coated with 1:50 diluted hESC‐qualified Matrigel (Corning, 354277) and cultured in mTeSR1 medium (STEMCELL Technologies, 85850), or coated with recombinant vitronectin human protein (Gibco, A14700) at a concentration of 0.5 µg/cm^2^ and cultured in Essential 8 Flex medium (Gibco, A2858501), in a humidified incubator under 5% CO2 at 37°C. The medium was replenished every other day or every day depending on cell confluency. Cells in both media conditions were regularly passaged when they reached 80%–90% confluency with TrypLE Express (Gibco, 12605010), usually 3–4 days at a ratio of 1:8–1:10. When passaged, the hESCs were washed with PBS for twice, then followed by digesting with TrypLE Express for 5 min to dissociate the clones into single cells. Cells were seeded onto Matrigel‐coated or vitronectin‐coated surfaces in mTeSR1 or E8 supplemented with 10 µm Y‐27632 (SELLECK, S1049). Twenty‐four hours after passaging, the medium was replenished with fresh mTeSR1 or E8 medium without Y‐27632. Culture effect studies were performed by culturing hESCs in E8 followed by mTeSR1 for five passages.

### 5.4 Organoids Differentiation of hESCs

The previously described protocol to differentiate hESCs into skin organoids were used [22]. In brief, colonies of hESCs were detached from the culture dishes using TrypLE Express. Once dissociated, the appropriate volume of single‐cell suspension was transferred into E8 or mTeSR1 medium containing Y‐27632 (20 µm). For centrifugation method, cells were distributed at a count of 3,500 cells in 100 µl per well into 96‐well U‐bottom plates (Thermo Fisher Scientific, 174925) and aggregated by centrifugation at 200 g for 5 min. For hanging drop protocol, 35 µl droplets of cell‐containing culture medium with 3,500 cells were placed on the inside of the lid of the 100 mm cell culture dish. The lid was then inverted onto the base of a dish containing 5 mL of PBS to allow hanging drop and cell aggregates formation. These cell aggregates were incubated in a humidified incubator under 5% CO2 at 37°C. After 24 h, fresh 100 µl medium was added to each well to dilute out Y‐27632 (10 µm) and promote cell proliferation and aggregate growth. To start differentiation (day 0), all cell aggregates were transferred to new 96‐well U‐bottom plates in 100 µl differentiation medium containing E6 (Gibco, A1516401), 2% Matrigel (Corning, 356230), 10 µm SB431542 (Selleck, S1067), 4 ng/ml bFGF (Millipore, GF003) and 2.5 ng/ml BMP4 (PeproTech, 120‐05). After 3 days, 25 µl of differentiation medium containing 250 ng/ml bFGF and 1 µm BMP inhibitor LDN193189 (Selleck, S2618) were added to each well, bringing the total volume to 125 µl. On day 6, 75 µl of fresh E6 medium was added, thus bringing the final volume to 200 µl. On days 8 and 10, half of the medium was removed and 100 µl fresh E6 medium was added. On day 12, all aggregates were transferred into individual wells of 24‐well low‐attachment plates (Thermo Fisher Scientific, 174930) in 500 µl organoid's maturation medium (OMM) containing 1% Matrigel (OMM1%M), and then the plates were placed on an orbital shaker for shaking at 80 rpm. OMM is composed of 1:1 of Advanced DMEM/F‐12 (Gibco, 12634010) and Neurobasal (Gibco, 21103049), 1 × GlutaMax (Gibco, 35050061), 0.5 × B27 minus vitamin A (Gibco,12587010), 0.5 × N2 supplements (Gibco, 17502048), 0.1 mm 2‐mercaptoethanol (Gibco, 21985023) and 1% Matrigel. Three days later, half of the spent medium was removed and 250 µl fresh OMM1%M was added. Starting from day 18, half of the medium was replenished every two or three days with fresh OMM without Matrigel, including a full medium change once a week starting from day 45. For sample collection, these organoids were rinsed with PBS for three times, then either flash‐frozen for RNA‐seq experiments or transferred to a 35 mm cell culture dish containing 4% PFA solution for immunohistochemistry.

### 5.5 Neural Differentiation of hESCs

hESCs colonies were disaggregated using TrypLE Express for 5 min, washed using hESCs medium and plated as monolayer culture on Matrigel‐coated dishes at a density of 10 000–25 000 cells/cm^2^ in mTeSR1 with 10 µm Y‐27632. After 24 h, the Y‐27632 was withdrawn, and neural induction was initiated by switching from hESCs growth medium to differentiation medium (DMEM/F‐12 supplemented with 1% N2, 1% B27 (Gibco, 17504044), NEAA (Gibco, 11140050), 20 ng/ml EGF (Peprotech, AF‐100‐15‐100UG), and 20 ng/ml bFGF) and cultured for 7 days with medium changes at 2–3 days intervals.

### 5.6 Pseudovirus Transfection

This SARS‐CoV‐2 pseudovirus carries both GFP and Luciferase reporter genes, allowing infection efficiency to be assessed by either fluorescence signal or luciferase activity. The specific process of organoids infecting SARS‐CoV‐2 pseudovirus (Yeasen Biotechnology, 11991ES50) is carried out according to the instruction manual. Briefly, organoids were cultured in a 24‐well ultra‐low attachment plate (Thermo Fisher Scientific, 174930) with 0.5 mL of medium per well containing SARS‐CoV‐2 pseudovirus on an orbital shaker rotating at 80 rpm. After virus exposure for 6 h, organoids were washed 3 times with fresh medium and returned to culture. 48 h post‐transfection, organoids were stained with GFP and AQP1, then captured using Nikon‐AXR confocal microscope.

### 5.7 Immunoblotting

Cells were lysed with RIPA lysis buffer (Beyotime, P0013B) with proteinase inhibitor cocktail (Beyotime, P1010) and Phosphatase inhibitor cocktail (Beyotime, P1086) for 20 min in ice and centrifuged at 10 000 g at 4°C for 10 min. Supernatants were collected and mixed with 5 × SDS‐PAGE loading buffer (250 mm Tris‐Hcl [pH 6.8], 10% SDS, 0.5% BPB, 50% glycerinum, 5% β‐mercaptoethanol) and heated to 100°C for 10 min. After electrophoresis, protein samples were transferred to PVDF membrane, which was blocked with blocking reagent (Roche, 11096176001) for 60 min followed by incubation with primary antibodies and second antibodies, respectively. Images were captured using a Protein Simple FluorChem system. Each experiment was carried out in triplicate, and a representative blot is shown unless otherwise stated. Uncropped and unedited Western blots are provided in Figure S7.

### 5.8 Immunofluorescent Staining

The monolayer cells were fixed with 4% (w/v) paraformaldehyde (PFA) for 15 min at room temperature, then permeabilized for 10 min with 0.2% TritonX‐100 and blocked for 60 min or overnight at 4°C with 3% bovine serum albumin (BSA) in PBS. Primary antibodies diluted in blocking buffer were applied to samples and incubated for overnight at 4°C. Samples were washed three times with PBS containing 0.01% Tween‐20 (v/v) (PBST), followed by incubation with fluorescently conjugated secondary antibodies diluted in blocking buffer for 2 h at room temperature. Samples were washed three times with PBST. Finally, cells were counterstained with 300 nm 4′,6‐diamidino‐2‐phenylindole (DAPI) solution at room temperature for 10 min.

C57BL/6 mice were purchased from Beijing Vital River Laboratory Animal Technology. Mice were euthanize and the brains were collected. The whole brains or organoids were placed directly in 4% PFA overnight at 4°C on a rocking shaker. After fixation, organoids were washed in PBS and cryoprotected by overnight incubation in 30% sucrose in PBS at 4°C. Organoids were placed in a plastic cryomold and embedded in gelatin, then snap frozen on dry ice. Frozen tissues were stored at −80°C until processing. Serial tissue sections (10 µm for organoids) were sliced using a cryostar (Thermo Fisher Scientific, NX50) and dried at room temperature, then stored at 20°C until ready for processing. For immunofluorescence staining, the sections were washed with PBST and permeabilized with 0.1% Triton X‐100 (v/v) for 10 min at room temperature. For antigen retrieval, sections were boiled in a 10 mm sodium citrate buffer (pH = 6) at 90°C for 10 min using a water bath. Slides were cooled to room temperature and washed with PBS containing 0.01% TritonX‐100 for 10 min. Blocking (5% BSA in PBS) for 1 h at room temperature followed by overnight primary antibody incubation at 4°C. After washing in PBS, tissue sections were incubated with secondary antibodies for 2 h at room temperature. After washing with PBS, slides were mounted in mounting solution, coverslipped, and sealed with nail polish, and imaged in a Nikon‐AXR confocal microscope.

The primary antibodies and appropriate fluorohore‐conjugated secondary antibodies used in this study are as fellows: OCT4 (Santa cruz, sc‐5279), SOX2 (Santa cruz, sc‐365823), NANOG (Thermo Fisher Scientific, PA1‐097X), PAX6 (Abcam, ab105405), AQP1 (Santa cruz, sc‐25287), NESTIN (HUABIO, HA722919), SOX1 (R&D Systems, AF3369), A2B5 (R&D Systems, MAB1416), TTR (R&D Systems, MAB7505), TTR (Proteintech, 11891‐1‐AP), ACE2 (Abcam, ab15348), GFP (Proteintech, 50430‐2‐AP), LUM (HUABIO, ET7109‐24), COL1A1 (Abcam, ab138492), CLDN3 (HUABIO, ER63024), KRT15 (Abcam, ab52816), ERBB2 (Abcam, ab134182), SORBS2 (Proteintech, 24643‐1‐AP), Calnexin (Proteintech, 81938‐1‐RR), BMP4 (Abcam, ab124715), GM130 (Cell Signaling Technology, 12480T), β‐catenin (Cell Signaling Technology, 19807S), GAPDH (Proteintech, 60004‐1‐Ig), Goat anti‐Mouse IgG (H+L) (Thermo Fisher Scientific, A‐11001), Goat anti‐Rabbit IgG (H+L) (Thermo Fisher Scientific, A‐11012).

### 5.9 LipidTOX Staining

LipidTOX Staining (Invitrogen, H34475) was performed to visualize sebaceous glands and lipid‐rich adipocytes according to manufacturer's protocol. Cryosections of skin organoids were incubated with LipidTOX neutral lipid stain diluted at a ratio of 1:500 in buffer to make a 1 × solution for 30 min at room temperature, followed by 300 nM DAPI solution at room temperature for 5 min to visualize nuclei. Images were then captured using Nikon‐AXR confocal microscope.

### 5.10 Purification of iCSF and Exosomes by Differential Centrifugation

For iCSF isolation experiments, conditioned media were collected and centrifuged at 500 g for 5 min to remove dead cells and debris, and subsequently analyzed via mass spectrometry. For Exosomes isolation experiments, conditioned media were sequentially spun (300 × g for 10 min, 2000 × g for 20 min, 10 000 × g for 30 min) to remove cells, cell debris, apoptotic body, respectively. Next, the suspension was ultracentrifuged at 100 000 × g for 70 min at 4°C and the supernatant was discarded. The remaining pellet was suspended in PBS and ultracentrifuged at 100 000×g for 70 min at 4°C again. The Exosomes pellet was then re‐suspended in 100 µl PBS for further characterizations.

### 5.11 Mass Spectrometry Analysis

After supplemented with 1% protease inhibitor, the protein concentration of iCSF and Exosomes were determined with BCA kit according to the manufacturer's instructions (Beyotime Biotechnology, P0011). A total of 100 µg of each protein sample was subjected to enzymatic hydrolysis and proteomics screening was accomplished by mass spectrometry analysis on a MALDI‐TOF‐MS instrument (Bruker Daltonics).

### 5.12 Transmission Electron Microscopy (TEM)

To visualize the ChP cells, ChP organoids were fixed in centrifuge tube with 2.5% glutaraldehyde and 2% paraformaldehyde in 0.1 M sodium cacodylate buffer overnight at 4°C. The following day, Samples were rinsed three times with PBS and postfixed with 1% osmium tetroxide in PBS on ice and in the dark for 1 h, washed five times over one hour with distilled water, and dehydrated in an ascending acetone series (30%, 50%, 70%, 80%, 90%, 95%, 100%). Then, the dehydrated samples were infiltrated and embedded in Epon‐812 Araldite epoxy resin. Pale gold 60–90 nm ultrathin sections were cut from the embedded samples using an ultramicrotome, then the sections were adsorbed on the surface of a carbon‐coated copper grid and stained with 1% uranylacetate for 20 min and lead citrate for 2 min. The coated side of the grids were left to dry before imaging on the electron microscope (JEOL, JEM‐1400FLASH).

To visualize Exosomes by TEM, Exosome pellet was resuspended in 50–100 µl of sterile filtered PBS. For each sample preparation, intact Exosomes (5 µl) were dropped onto carbon‐coated 400 hex mesh copper grid washed with sterile filtered PBS and stained with 1% uranyl acetate. Finally, grids were imaged by transmission electron microscopy.

### 5.13 Quantitative Real‐Time qPCR Analysis

Total RNAs were extracted using the RNAsimple Total RNA Kit (TIANGEN, DP419). cDNA was synthesized using PrimeScriptRT reagent Kit (Takara, RR037A), and then amplified with SYBR green master mix (Takara, RR820B) by specific primers. GAPDH was used as an internal control, and signals in each sample were normalized against it. Primer sequences are listed in Table S6.

### 5.14 RNA Sequencing, Library Preparation, and Analysis

Total RNAs were extracted from hESCs using TRIzol Reagent (Invitrogen, cat. NO 15596026) according to manufacturer's protocol. DNA digestion was carried out after RNA extraction by DNase I. RNA quality was determined by examining A260/A280 with Nanodrop Onec spectrophotometer (Thermo Fisher Scientific). RNA Integrity was confirmed by 1.5% agarose gel electrophoresis. Qualified RNAs were finally quantified by Qubit3.0 with QubitTM RNA Broad Range Assay kit (Life Technologies, Q10210).

2 µg total RNAs were used for stranded RNA sequencing library preparation using KC‐DigitalTM Stranded mRNA Library Prep Kit for Illumina (Catalog NO. DR08502, Beijing Novogene Co., Ltd. China) following the manufacturer's instruction. The kit eliminates duplication bias in PCR and sequencing steps, by using unique molecular identifier (UMI) of 8 random bases to label the pre‐amplified cDNA molecules. The library products corresponding to 200–500 bps were enriched, quantified and finally sequenced on DNBSEQ‐T7 sequencer (MGI Tech Co., Ltd. China) with PE150 model.

Raw sequencing data was first filtered by Trimmomatic (version 0.36), low‐quality reads were discarded and the reads contaminated with adaptor sequences were trimmed. Clean Reads were further treated with in‐house scripts to eliminate duplication bias introduced in library preparation and sequencing. In brief, clean reads were first clustered according to the UMI sequences, in which reads with the same UMI sequence were grouped into the same cluster. Reads in the same cluster were compared to each other by pairwise alignment, and then reads with sequence identity over 95% were extracted to a new sub‐cluster. After all sub‐clusters were generated, multiple sequence alignment was performed to get one consensus sequence for each sub‐clusters. After these steps, any errors and biases introduced by PCR amplification or sequencing were eliminated.

For organoids RNA‐sequencing, to avoid overlooking rare but functionally important cell types, we did not pool all cells together from the ChP organoids for RNA‐seq. Instead, the organoids were dissected at the junction, and then the two portions were subjected to RNA‐seq separately. Organoids were pooled such that for one biological replicate, 6 organoids were used, and hESCs was used as a control.

### 5.15 Statistics and Reproducibility

All the statistical data are presented as the mean ± SD, unless indicated otherwise. Each test was repeated at least three times, and the results were analyzed by GraphPad Prism 8 software. Statistical analysis was performed using Student's t‐test. p values <0.05 were considered statistically significant. All the experiments were conducted at least three times unless indicated otherwise.

## Author Contributions

ZLC, LXJ, RCL, MS, LZC and PL designed the experiments. ZLC and LXJ performed most of the experiments. XBW performed the organoids RNAseq analysis. LLZ performed the mouse Immunohistochemistry. RCL and MS participated in the data analysis and discussion of the study. RCL, MS, LZC and PL wrote the paper. All authors read and approved the final manuscript.

## Conflicts of Interest

The authors declare no conflicts of interest.

## Supporting information




**Supporting File 1**: advs77512‐sup‐0001‐SuppMat.docx.

**Supporting File 2**: advs77512‐sup‐0002‐S1.xlsx.

**Supporting File 3**: advs77512‐sup‐0003‐S2.xlsx.

**Supporting File 4**: advs77512‐sup‐0004‐S3.xlsx.

**Supporting File 5**: advs77512‐sup‐0005‐S4.xlsx.

## Data Availability

The data that support the findings of this study are available from the corresponding author upon reasonable request.
