# Supplementary material for: Niche‐Mediated Neural Priming Enables Robust and Scalable Generation of Human Choroid Plexus Organoids
Source: Adv Sci (Weinh). 2026 Aug 31:e77512. Online ahead of print. doi: 10.1002/advs.77512 (PMC13530365; doi:10.1002/advs.77512)
Supplement: Supplementary file 1 [file ADVS-9999-e77512-s002.docx]

**Niche-mediated Neural Priming Enables Robust and Scalable Generation of Human Choroid Plexus Organoids**

Zhongliang Chen^1,2#^, Liangxia Jiang^3,4#^, Xianbin Wang^5,6^, Lili Zhong^7^, Rongcan Luo^8*^, Min Su^1*^, Liangzhao Chu^2*^, Peng Luo^9,10*^

**Figure S1. Generation of sporadic ChP organoid-like aggregates during skin organoid induction. A,** Morphologies of developing skin organoids during differentiation. Representative brightfield (d0-d36) and Darkfield images (d102) of skin organoids derived from H9 hESCs. Lower right**,** Representative brightfield (d44) of ChP organoids derived from H9 hESCs. Scale bars, 500 µm. **B-C,** LipidTOX (green) staining of day 102 skin organoids reveals lipid-rich cells such as sebaceous glands and adipocytes. Immunostaining for KRT15 (red) and AQP1 (green) labels the outer root sheath of hair follicle cells and choroid plexus epithelium, respectively. Cell nuclei are stained blue with DAPI. Arrows highlight a hair shaft (white) and a sebaceous gland (yellow), respectively. Scale bars, 500 μm; BOX, scale bars, 100 μm. **D,** Immunostaining for TTR (red) and AQP1 (green) in the aggregates and skin organoids. Cell nuclei are stained blue with DAPI. Scale bars, 250 μm; BOX, scale bars, 100 μm. **E**, RT-qPCR analysis shows that the expression of the skin marker KRT15, ChP markers *TTR* and *AQP1* in the ChP organoids and skin organoids.

**Figure S2. iCSF does not contain components of *in vivo* CSF. A**, Morphologies of developing ChP organoids derived from H1 hESCs. Scale bars, 500 μm. **B**, Representative confocal images of fluorescent staining for ChP epithelium markers TTR (red) and AQP1 (green) in ChP organoids derived from H1 hESCs at day 38. Cell nuclei were stained blue with DAPI. Scale bars, 250 μm. **C**, Heat map plotted from normalized read counts shows an overall upregulation of transporter genes (belonging to the Slc gene family) in day 45 ChP organoids. Color bars: blue (low expression), red (high expression). **D**, gProfileR analysis of proteins detected only in *in vivo* CSF samples and histogram showing top categories from gProfileR analysis with significant (p<0.05) enrichment (p<0.05) for GO categories cellular component (GO:CC), molecular function (GO:MF) and biological process (GO:BP), KEGG, REAC, WP databases. **E**, Venn diagram showing overlap of proteins identified in iCSF and iCSF-derived exosomes.

**Figure S3. ChP organoids may represent a mixture of ChP from different ventricles. A**, Heat map showing the differential expression the specific markers of different ventricles ChP between hESCs and ChP organoids. **B**, RT-qPCR analysis of the expression of some genes shown in (**A**). **C**, Barplot showing the absolute expression, measured by RNAseq, of different ventricles ChP between ChP organoids and hESCs.

**Figure S4. ChP organoids are Susceptible to SARS-CoV-2 Infection. A**, Heatmap comparing expression of SARS-CoV-2 receptor genes *ACE2*, *NRP1* and *TMPRSS2* between hESCs and ChP organoids. **B,** RT-qPCR analysis of the expression of the genes shown in **(A). C**, Morphologies of developing ChP organoids (ChP’O) and Brain organoids (Brain’O) during differentiation. Representative brightfield (left) and HE staining image (middle) of these two organoids derived from H9 hESCs. By day 35 of differentiation, the ChP organoids typically form epithelial structure, whereas the Brain organoids encompass many neural tube-like structure. Scale bars, 500 µm. **D**, Representative confocal images of fluorescent immunohistology for TTR (red), and SOX2 (green) in ChP organoids and Brain organoids at day 35. Cell nuclei were stained blue with DAPI. Scale bars, 500 μm. **E**, Immunoblotting for neuron marker βⅢ TUBULIN, ChP markers AQP1 and TTR in ChP organoids and Brain organoids. **F**, RT-qPCR analysis for the expression of neuron marker *DCX* and *FOXG1*, ChP markers *AQP1*, *HTR2C* and *TTR* in ChP organoids and Brain organoids. **G**, Representative confocal images of fluorescent immunohistology for AQP1 (green), ACE2 (red) in ChP organoids and Brain organoids. Cell nuclei were stained blue with DAPI. Scale bars, 200 µm. **H**, Representative confocal images of fluorescent immunohistology for SARS-CoV-2 spike pseudovirus infection of ChP organoids and Brain organoids. GFP-positive cells were detected by GFP antibody, Nuclei in blue are stained with DAPI. Scale bars, 200 µm.

**Figure S5. Switching from E8 to mTeSR1 Medium Enhances the Neural differentiation potential of hESCs. A**, Immunofluorescent for pluripotent stem cells markers NANOG (red) and OCT4 (green) in E8 and mTeSR1 cultured hESCs. Cell nuclei were stained blue with DAPI. Scale bars, 200 µm. **B**, RT-qPCR analysis of the expression of pluripotent stem cells markers *SOX2*, *NANOG* and *KLF4* in E8 and mTeSR1 cultured hESCs. **C**, *left*: Immunoblotting for β-catenin, BMP4, ERBB2, SORBS2 and OCT4 in hESCs cultured in E8 and mTeSR1 medium. *right*: quantification of band intensities of these proteins in three independent experiments. **D**, Altered expression of functionally related genes of neural system development detected by RNA-seq analysis of mTeSR1 culture. **E**, Top 20 of upregulated KEGG enrichment identified in mTeSR1 culture.

**Figure S6. The ability of E8 and mTeSR1 cultured hESCs to form aggregates is different. A**, Aggregates formation by using the methods of hanging drop or centrifugation of E8 and mTeSR1 cultured hESCs. Representative images of brightfield (left), hanging drop (middle), and centrifugation (right). Scale bars, 500 µm. **B**, Top 30 of GO enrichment identified in hESCs cultured with E8 medium. **C**, Immunofluorescent for pluripotent stem cells markers NANOG (red) and OCT4 (green) in aggregates derived from E8 and mTeSR1 cultured hESCs. Cell nuclei were stained blue with DAPI. Scale bars, 250 µm.

**Figure S7. Unedited and uncropped Western blots**

**Supplementary tables**

**Table S5. The top 100 proteins listed in the ExoCarta database are mostly found in iCSF exosomes.**

| **The top 100 proteins listed in the ExoCarta database** | | | |
| --- | --- | --- | --- |
| **Exist in iCSF exosomes** | | **Do not exist in iCSF exosomes** | |
| **Proteins** | **Number** | **Proteins** | **Number** |
| CD63 CD9 TSG101  PDCD6IP CD81 GAPDH  FLOT1 SDCBP ANXA2  ACTB HSP90AA1 ITGB1  VCP LDHA PGK1  ALDOA HSP90AB1 EEF1A1  ENO1 HSPA8 ANXA1  PPIA PKM FLNA  ANXA5 HSPA1A EEF2  YWHAZ FASN MSN  SLC3A2 YWHAE RAP1B  LGALS3BP ANXA6 PRDX1  CLTC CFL1 TPI1  PFN1 ACTN4 MVP  ATP1A1 YWHAQ LDHB  GNB2 GNAI2 HSPA5  CLIC1 CDC42 CCT2  PRDX2 EPRS CD44  YWHAG TFRC MYH9  KPNB1 FN1 RAB5C  EZR BSG ANXA4  EHD4 YWHAB GDI2  ANXA11 ACTN1 FLOT2  CCT5 UBA1 RAN  RAB7A RAB14 GNB1  FLNB CCT3 RHOA  MFGE8 ACLY MYO1C  EHD1 ATP5B TCP1  SLC16A1 RDX HGS  GPI EEF1G ANXA7  AHCY TUBB4B THBS1 | 93 | CD274  HIST1H4A  ITGA6  LAMP1  ARF1  TUBA1C  TKT | 7 |

**Table S6 Primers used in this study.**

| **Gene** | **Forward** | **Reverse** |
| --- | --- | --- |
| ACE2 | TTCGATATTACACAAGGACCC | TCTGTCCAGCTTCTGTAGAG |
| AQP1 | CCTCCTGGCTATTGACTACA | AGTCGTAGATGAGTACAGCC |
| ARC | ACGCAGATCTTCGAGGACC | CATGTGATTCTGGATCTGGG |
| BMP4 | AACCGAATGCTGATGGTCGT | AGAAGTGTCGCCTCGAAGTC |
| BMP6 | GCTGCCAATTACTGTGATGG | CAGCACGGTTTGGGGACATA |
| BMP7 | ATGTTCATGCTGGACCTGTA | TGAGGAAATGGCTATCTTGCA |
| CLDN1 | CTTTGACTCCTTGCTGAATCT | CCAAGCACTTCATACACTTCA |
| CLDN2 | TGATGGTGACATCCAGTGCA | AAAAGACTCCACCTGCTACC |
| CLDN3 | AGTGCAAGGTGTACGACTCG | TGTCGTCCTGCACGCAGTT |
| CLDN4 | AAGACTTCTACAATCCGCTGG | GTGGACAGTTGCAGCAAAGC |
| CLDN4 | TTCGTTGCGCTCTTCGTGAC | GTTGGCGAACCAGCAGAGT |
| CLDN5 | TTCGTTGCGCTCTTCGTGAC | GTTGGCGAACCAGCAGAGT |
| CLIC6 | CACGAAGAAGGATGCAAATGA | CAGAAACAGTGACATCCTCG |
| CNTN2 | GAGACCCAGTGAAAGCTCAT | GAAGTTGGGGAACTCGTTGA |
| COL1A1 | CTCCTGGCAAAGATGGACTC | GAAGCTGAAGTCGAAACCAG |
| DBNL | GGAGCCTGAGTGCATCATG | TCAGACACGGCATTGGTCTT |
| DCN | CCGTTTCAACAGAGAGGCTT | CTCGAAGATGGCATTGACAG |
| EMX2 | TCATCCACCGCTACCGATAT | GCGTGTTCCAGCCTTAGAAG |
| ENC1 | GAAATAGGACCTTCCCTTGC | TGGGTGGATGGAATTGTCAAA |
| ERBB2 | ACAACACAGACACGTTTGAGT | CGTCCGTAGAAAGGTAGTTG |
| FAM166B | CTGCCCACTACTTCGGTTCA | AGGTAGACTCTCCCTGGGAA |
| FHIT | TCGCTCTTGTGAATAGGAAAC | ACTCTCTGGGTCGTCTGAAA |
| GAPDH | GCCTCAAAATCCTCTCGTTGTG | GGAAGATGGTGATGGGATTTC |
| HESX1 | TGAGAGAATCTTAGGACTGGA | AGGATTTGGGACATGTAGACA |
| HTR2C | TCATGAAGATTGCTATTGTTTG | GAAGGACCCAATAAGAACGAA |
| ID1 | AACGGCTGTTACTCACGCC | GATTCCGAGTTCAGCTCCAA |
| ID4 | GAGATCCTGCAGCACGTTAT | CGGTGTTGAGCGCAGTGA |
| Lefty1 | CAAAGAGGTGCCCACCCTG | TGTGTGCTGGCCTCCAACG |
| Lefty2 | AGCGAGGTGCCCGTACTG | GCTCTGGCTGAACCTCTTTC |
| LUM | GTGGTACCAGTGGCCAGTA | CAGTACATGGCACTTGGGTA |
| LY6E | CAACTACTGCGTGACTGTGT | GAAGCCACACCAACATTGAC |
| MT3 | TGCCCTTCTGGTGGCTCCT | TTGGCACACTTCTCACACTC |
| NODAL | TCGACATCACTTGCCAGACA | CAACAGGATTAGGACACTCG |
| NRP1 | CCCCAAACCACTGATAACTCG | AGACACCATACCCAACATTCC |
| PENK | ATGAGGAAGAAGTGAGCAAGA | GTAGTCCATCCACCACTCTG |
| SIX3 | GAAGAGTTGTCCATGTTCCAG | GACTCGTGTTTGTTGATGGC |
| SMAD3 | ACTGGACGACTACAGCCATT | CACTGGTTTCTCCATCTTCAC |
| SOD3 | CTACTGTGTTCCTGCCTGCT | GTACATGTCTCGGATCCACT |
| SORBS2 | CACCAACTCAGAGCCAGATA | GACAATGCTGGAGTTGAGTG |
| TMPRSS2 | GGAGTGTACGGGAATGTGATG | CCAGCCCCATTGTTTTCTTG |
| TTBK1 | TGTGTGCAGGTTCATTGGCT | AATGTGGTGCTCAGCGTGAA |
| TTR | TCAAAGTTCTAGATGCTGTCC | TCCACTTTGTATATCCCTTCT |
| WNT3 | CAATTACATCGAGATCATGCC | GTCATCTATGGTGGTGCAGT |
| WNT4 | ACAGATGAGGACCTGGTGTA | TTGGACGTCTTGTTGCATGT |
| WNT5A | CTTCGCCCAGGTTGTAATTG | TGGTCCTGATACAAGTGGCA |
| WNT5B | TTCTGACAGACGCCAACTC | CTCCTGGTACAATTGGCACA |
| DCX | TGGATTCATTTACAACTGTTAG | TGTCCAAAATCAAGTTCCATAT |
| FOXG1 | CAGCACTTTGAGTTACAACG | GTGGAGAAGGAGTGGTTGTT |
| KLF4 | CTCTCCCACATGAAGCGACT | TTGAACTCCTCGGTCTCTCT |
| NANOG | TCTGCTTATTCAGGACAGCC | CATTGAGTACACACAGCTGG |
| NKCC1 | CGAGATGCCACTGGAAACGT | CGAGATGCCACTGGAAACGT |
| SOX2 | TGTCCCACTGCTGATTATTGA | AAGGTAAGCTTCCCAACGAC |

**Description of Table S1-S4 (separate file).**

**Table S1. Mass spectrometry data of iCSF (day 35 and day 50) and Fresh Medium.** emPAI value for each protein detected.

**Table S2. The top 100 proteins of iCSF are mostly found in human embryonic, pediatric, and adult CSF.** CSF data from embryonic human (30), pediatric human (31) and adult human (29) compared with proteins detected in iCSF.

**Table S3. ChP organoids secrete human disease-related biomarkers.** Table showing the biomarkers detected in iCSF and/or exosomes, and with average emPAI>1, the disease or condition in which the biomarker levels are altered and the relevant literature.

**Table S4. Mass spectrometry data of Exosomes derived from iCSF (day 50).** emPAI value for each protein detected.
